# Supplementary material for: Quasi-experimental evaluation of national border closures on COVID-19 transmission
Source: PLOS Glob Public Health. 2023 Feb 28;3(2):e0000980. doi: 10.1371/journal.pgph.0000980 (PMC10021705; doi:10.1371/journal.pgph.0000980)
Supplement: S1 Text — (PDF) [file pgph.0000980.s001.pdf]

Supplementary Information

Data Collection and Preparation

Outcomes

We used a five-day lag in R_t in our primary analyses to represent the typical incubation period of the disease [1]. Other outcome measures were considered but deemed inappropriate for this analysis; the availability of excess mortality data was limited in low- and middle-income countries, while modelled outcomes lack the structural breaks needed for quasi-experimental designs [2]. To further evaluate the validity of our primary outcome measure, we examined whether changes in testing capacity were associated with changes in R_t over the study period (Fig A in S1 Text). Since the rapid increase in global testing capacity in the early months of COVID-19's first wave gradually reduced incidence underestimation, national and global R_t estimates should not be considered true parameters. However, because fluctuations in global testing capacity do not appear to be systematically associated with the implementation of total or targeted border closures (Fig 1), quasi-experimental methods can be used to identify longitudinal breaks in the transmission of the pandemic [3]. We also provide analyses using different lagged outcomes between zero and 15 days in the appendix tables to investigate the impact of delays in data reporting, the time needed for second-order transmission to occur, and the delay introduced by a sliding time window used to calculate R_t.

We used the EpiEstim 2.2 R software package to estimate R_t as a measure of COVID-19 propagation based on daily incidence of COVID-19 as compiled by the Center for Systems Science and Engineering (CSSE) at Johns Hopkins University [4–7]. A prior mean value for R_t of 2.25 (S.D. 1) and serial interval of 4.7 (S.D. 2.9) was used to produce estimates over a 3-day sliding time window for each time series [5,8–10]. Separate time series of R_t were calculated for global incidence (daily sums of all countries, both including and excluding China), for individual countries, and for high income countries (HICs) and low- and middle-income countries (LMICs), both including and excluding China.

Border Closure Coding

For the border closure coding, we hand-coded matrices of border closures represented as dichotomous variables by date of implementation and country targeted for 179 countries using data from the Oxford COVID-19 Government Response Tracker, with corrections, updates and additions detailed in S1 Data [11]. This matrix was then used to code categorical indicators of the daily travel restriction status between all country-pairs: 1) *no border closure* – the country has not implemented any closure of land, air, or sea border in response to the COVID-19 pandemic; 2) *targeted border closure* – the country restricts non-essential entry of foreign nationals from one or more specified countries; 3) *total border closure* – the country restricts all non-essential entry of foreign nationals; or 4) *reopening* – the country had previously implemented a total border closure and has re-opened borders to at least one country.

Visa suspensions and closure of land borders were coded uniquely as de facto border closures and analyzed as targeted border closures in quantitative analyses. Eleven jurisdictions (Aruba, Barbados, Bermuda, Cabo Verde, Greenland, Guam, Hong Kong, Kosovo, Macao, and Puerto Rico, and Solomon Islands) were excluded due to low data availability in either outcome or covariate data and to limit analysis to countries. Two countries, North Korea and Turkmenistan, reported no cases and were not included in analyses. China was also exempted from primary quantitative analyses because, as the first country to report cases of COVID-19 and as the initial epicenter of the pandemic, national border closures could not have controlled

domestic transmission in this study period. The final dataset contains border closure data for 179 countries, and full outcome and intervention data for 166 countries.

Covariates

A full list of control variables hypothesized to have been associated with the effectiveness of border closures is available in Table A. All covariates were specified *a priori* and obtained from publicly available databases and were selected to cover categories of country characteristics, economic factors, gender parity, health indicators, policy control indicators, and border closure details. Where data was not available for the current year, we used data from the most recent year available. Further information is made available in a metadata repository alongside our open-access dataset on Scholars Portal Dataverse with download dates, updated data sources, and variable format and description (S1 Data).

Quantitative Methods

The proportion of the world's population targeted by border closures was calculated as the sum of the population of targeted countries divided by the population of all countries included in the study. A measure excluding own population was also calculated to account for the limited impact of border closures for countries with large populations. The proportion of global cases targeted by border closures was calculated as the sum of cumulative incidence for every country targeted up to the day being evaluated divided by the global cumulative incidence. The decision to limit our study to the first 22 weeks of the COVID-19 pandemic was driven by considerations related to our quasi-experimental methods as well as global border closure trends. Our analysis plan (S3 Data) was developed once our team determined that new border closures had begun to stall in May 2020, as observed in Fig 2. Because interrupted time series analysis can be performed on a balanced dataset with a minimum of 12 data points [12], adding a lag time of five days to the final country-specific border closure on May 27, 2020 led us to the 22-week mark. All calculations were conducted in Stata release 15 and maps created using Tableau version 2020.2 unless otherwise stated [13,14].

Interrupted Time-Series Analysis

Country-level intervention points were evaluated for all targeted and total border closures that met the following criteria: 1) a minimum of seven days of data exists prior to and after the intervention point, 2) for multiple intervention time series, a minimum of seven days has passed since the last intervention point, and 3) for multiple sequential targeted border closures, the second (or third) intervention represents an increase of at least 20% of the world's population being targeted by the new border closures. As robustness checks, Dickey-Fuller tests were run for every time series, and none exceeded the 5% critical value of the t-distribution for a unit root (S2 Data). Results correcting for serial autocorrelation were calculated using Cumby-Huizinga general tests for autocorrelation in time series analysis (S4 Data) [15].

We additionally considered alternate models aligning by the date of total border closures for both the global average and population-weighted global average of R_t. Stratified analyses of high-income and low- and middle-income countries (as classified by the World Bank), were conducted using pooled data with new intervention points calculated for each group exceeding the 20% global population threshold for both targeted and total restrictions, and the same analytical methods as global analyses [16].

Falsification tests were run to evaluate whether results were being driven by chance or analytical choices. A false intervention date was created at the midpoint between the first date 90% of the world's population was living in countries implementing border closures and the final date in the study period, and for each country that implemented only a total border closure, country-specific false intervention points were created at the midpoint between the date of total border closure and the final date in the study. In consideration of

the visually apparent peak in global R_t occurring between February 18-21, 2020, whose extreme values could skew findings, a robustness check was run dropping four values that exceed an R_t of 4.0. Finally, a robustness check restricting the length of time after the global total border closure intervention to match the period of time after global targeted border closure intervention (45 days) was conducted, with the global ITS results remaining unchanged (Tables G-H).

Meta-regression

The proportion of the global population and cases targeted by each restriction was calculated for the first targeted restriction for each country, and countries were categorized into three groups for the timing of restrictions relative to incidence within each country and relative to border closures implemented by other countries. Each country's first restriction, first targeted restriction, and first total restriction was categorized as happening either prior to 50, between 50 and 500, or after 500 cumulative domestic COVID-19 cases. Each country's first border closure, first targeted border closure, and first total border closure was also categorized as occurring within the first, second, or third tercile (third) of border closures globally. Every factor listed in Table A was first run against the sum of positive, negative, and null effects for targeted, total, and all border closures for both unlagged and five-day lagged outcomes and evaluated for significance at the 95% level (Table T). Full model regressions of every covariate found to have been independently associated with changes in R_t were then conducted. Robustness checks with unlagged outcomes produced similar results, but also identified higher GDP as being associated with beneficial total border closures and higher GDP per capita associated with less beneficial closures (Table W).

Fixed-Effects Time-Series Regression

Fixed effects regression was used to inform a robustness check for CEM analyses through identifying country-level indicators which significantly modified the association between border closures and the transmission of COVID-19. The primary evaluations used a five-day lag in R_t and after surveying published literature, a list of country characteristics was organized into a theoretical framework, including country-level indicators for the economy, gender, health, and domestic containment and closure policies (Table A). Within the fixed-effects regression model, the index i represents the individual country $(i = 1, \ldots, 166), t$ is the day of the study period between Jan 1 to June 8, 2020 $(t = 1, \ldots, 160)$, and x_{it} is the vector of independent variables evaluated for association (Equation 1). For the primary panel analysis, each of the twenty-one country-level indicators $x_{it} = [x_a, \ldots, x_m]$ were independently interacted with the percentage of global population targeted (excluding own population).

A fixed-effects regression model mitigates for potential confounding by controlling for country-level observables, while the model assumptions also accounted for the unobservable country-level factors.[17] The additional use of interaction effects examined the intra-country association between the vector of independent variables x_{it} and R_t outcomes as related to the intra-country variation observed in changes in the percentage of global population targeted (excluding own population). Nineteen country-level factors were identified at a significance level of 90% (Table AH). Scenario analyses using the percentage of global cases targeted (excluding own cases) as the intervention variable and R_t values lagged by zero, 10 and 15 days were also conducted (Table AI).

$$R_t = \beta_0 + \beta * x_{it} * Poptarget_{it} + u_{it}, \qquad with\ x_{it} = [x_a, \ldots, x_m] \qquad (Eq.\ 1)$$

Coarsened Exact Matching

Coarsened exact matching (CEM) was used to match and reduce the multivariate imbalance within countries with no border closure (control group) and countries with a border closure (treatment group). [18] An adjusted regression analysis using maximum likelihood estimation (MLE) was then fitted on the dataset with improved balance and used in predicting the size of the treatment effect averaged over all treated countries in our sample.[18]

The four border interventions of interest are: i) targeted border closures; ii) proportion of global population targeted by a targeted border closure; iii) proportion of global cases targeted by a targeted border closure; and iv) total border closures, with instances of border de-escalation censored from all CEM analyses. A diagrammatic representation of the coding of treatment status by type of border closure studied is provided in Fig B in S1 Text. To evaluate targeted border closures, the treatment group was comprised of all countries that had ever implemented a targeted border closure and the control group included countries that had never implemented a targeted border closure. In one model, data after a country escalated to a total border closure were censored for both treatment and control groups, while in another model, total border closure data were left intact and controlled for in MLE.

To evaluate total border closures, two approaches to assigning treatment status were used. The primary model assigned all countries that had ever implemented a total border closure to the treatment group and all countries that had never implemented a total border closure to the control group. A conservative model was also constructed, assigning countries that had *only* ever implemented a total border closure to the treatment group and all countries that had either never implemented a border closure, only implemented a targeted border closure, or escalated from a targeted border closure to a total border closure to the control group. The same two techniques outlined above for the targeted border analyses was used for both models in evaluating total border closures: censoring data during period of total border closure for both treatment and control groups or controlling for any instance of total border closures in MLE.

Due to the trade-off between matching on more information (a higher number of variables) and a reduction in the dataset size, varying degrees of coarsening were used in the matching process. Countries were matched on a subset of the independent variables identified to have significant interaction effects in the fixed-effects regression, with variations in the degree of coarsening. All country-day data points without a matched pair and a zero value for the CEM weight was excluded from further analysis.

The adjusted regression analysis used MLE to determine the average treatment effect on the treated in the newly balanced dataset. d_{it} and d_{jt} are dichotomous variables representing treatment status, with d_{it} indicating a country having implemented a targeted border closure (Eq 2) and d_{jt} indicating a country having implemented a total border closure (Eq 3). The coefficient β quantifies the average treatment effect across all countries having either implemented a targeted border closure or a total border closure.

$$R_t = \beta_0 + \beta * d_{it} * Poptarget_{it} + \alpha_i + u_{it} \qquad \textit{(Eq 2)}$$

$$R_t = \beta_0 + \beta * d_{jt} * Poptarget_{jt} + \alpha_j + u_{jt} \qquad \textit{(Eq 3)}$$

The primary analysis (Table 2) moderately coarsened variables with higher causal plausibility, and a robustness check minimally coarsened lower priority variable (Table Z). A robustness check on the primary model verified the improved balance after matching (Table AJ). Additional robustness checks using higher causal plausibility included greater coarsening (Table X) and minimal coarsening (Table Y). Furthermore, a robustness check to address concerns of confounding from changes in domestic containment and closure policies on the effects of border closures was conducted using moderate coarsening (Table AE). A robustness check was conducted using variables from the highest R-squared model with moderate coarsening (Table AK).

Further robustness checks coarsened by first limiting to the countries matched on in the primary analysis moderately coarsening on the closure policy variables and higher plausibility variables (Table AF), and then a follow-up robustness check matching only on closure policy variables (Table AG). More robustness checks included limiting by the initially matched countries and restricting the time period pre/post global intervention dates by forty-five days (Table AA) or by sixty days (Table AB). The length of forty-five days prior to the global targeted border closure is around the time when the first estimated case of COVID-19 was recorded, and about forty-five days later is the global total border closure date. The same period of 60 days was used in the total border closure analysis. Lastly, we conducted scenario analyses using a subset of the matched dataset and segregated by higher- or lower-tier covariate distributions, using the mean as the baseline for targeted border closures (Table AC) and for total border closures (Table AD). Regardless of the approach employed in selecting the number of variables used in the matching process and the level of coarsening, all results were highly consistent.

Limitations

There were a number of challenges to conducting a rapid evaluation of national border closures in the midst of the ongoing COVID-19 pandemic. Although there is no perfect source of information to quantify COVID-19 incidence, we strived to maximize comparability between countries, legitimacy of data sourcing, and completeness of information. The OxCGRT Data Repository was deemed to best satisfy these objectives, however, issues relating to testing, data reporting, and data comparability remain. Reported cases of COVID-19 are highly dependent on the degree of testing being done to detect transmission, and the accuracy and timeliness of data reporting.

Some challenges to coding the border closure matrix include data validation, reconciling unspecified countries by targeted closures, and missing country data. Firstly, due to the vast size of the OxCGRT dataset, we purposively verified the correctness of coding and cited news sources for 1) countries with large populations; 2) countries with early border closures; and 3) randomly selected countries for a final layer of accountability. Secondly, four countries targeted countries with greater than a stated number of cases but did not explicitly specify the countries to be targeted. We determined the list of countries targeted for these countries based on John Hopkins University case counts.[4] Lastly, seven countries with populations over 1 million that were missing from the OxCGRT dataset were added for analysis (Armenia, Equatorial Guinea, Guinea-Bissau, Latvia, North Macedonia, Timor-Leste, and Togo).

References

1. Lauer SA, Grantz KH, Bi Q, Jones FK, Zheng Q, Meredith HR, et al. The Incubation Period of Coronavirus Disease 2019 (COVID-19) From Publicly Reported Confirmed Cases: Estimation and Application. Annals of Internal Medicine. 2020 May 5;172(9):577–82.

2. Financial Times. Coronavirus excess mortality data [Internet]. Financial Times; 2020. Available from: https://github.com/Financial-Times/coronavirus-excess-mortality-data

3. To access the Appendix, click on the Appendix link in the box to the right of the article online.

4. Dong E, Du H, Gardner L. An interactive web-based dashboard to track COVID-19 in real time. The Lancet Infectious Diseases. 2020 May 1;20(5):533–4.

5. Thompson RN, Stockwin JE, van Gaalen RD, Polonsky JA, Kamvar ZN, Demarsh PA, et al. Improved inference of time-varying reproduction numbers during infectious disease outbreaks. Epidemics. 2019;29:100356.

6. Cori A, Ferguson NM, Fraser C, Cauchemez S. A New Framework and Software to Estimate Time-Varying Reproduction Numbers During Epidemics. Am J Epidemiol. 2013 Nov 1;178(9):1505–12.

7. Wallinga J, Teunis P. Different Epidemic Curves for Severe Acute Respiratory Syndrome Reveal Similar Impacts of Control Measures. Am J Epidemiol. 2004 Sep 15;160(6):509–16.

8. Nishiura H, Linton NM, Akhmetzhanov AR. Serial interval of novel coronavirus (COVID-19) infections. International Journal of Infectious Diseases. 2020 Apr 1;93:284–6.

9. Zhang S, Diao M, Yu W, Pei L, Lin Z, Chen D. Estimation of the reproductive number of novel coronavirus (COVID-19) and the probable outbreak size on the Diamond Princess cruise ship: A data-driven analysis. International Journal of Infectious Diseases. 2020 Apr 1;93:201–4.

10. World Health Organization. Report of the WHO-China Joint Mission on Coronavirus Disease 2019 (COVID-19) [Internet]. 2020 Feb [cited 2020 Jul 13]. Available from: https://www.who.int/docs/default-source/coronaviruse/who-china-joint-mission-on-covid-19-final-report.pdf

11. Hale T, Webster, Petherick A, Phillips T, Kira B. Oxford COVID-19 Government Response Tracker [Internet]. Blavatnik School of Government; 2020 [cited 2020 Apr 28]. Available from: https://www.bsg.ox.ac.uk/research/research-projects/coronavirus-government-response-tracker

12. Zhang F, Wagner AK, Ross-Degnan D. Simulation-based power calculation for designing interrupted time series analyses of health policy interventions. Journal of Clinical Epidemiology. 2011 Nov;64(11):1252–61.

13. StataCorp. Stata Statistical Software: Release 15. College Station, TX: StataCorp LLC; 2017.

14. Tableau Software, LLC. Tableau Desktop Release 2020.2. 2020.

15. Baum CF, Schaffer ME. ACTEST: Stata module to perform Cumby-Huizinga general test for autocorrelation in time series [Internet]. Statistical Software Components. Boston College Department of Economics; 2015 [cited 2020 Jul 13]. Available from: https://ideas.repec.org/c/boc/bocode/s457668.html

16. The World Bank. World Bank Open Data [Internet]. DataBank. Available from: https://data.worldbank.org/

17. Verbeek M. A Guide to Modern Econometrics. Chichester, England: John Wiley & Sons; 2008.

18. Iacus SM, King G, Porro G. Matching for Causal Inference Without Balance Checking. Available from: http://gking.harvard.edu/files/abs/cem-abs.shtml

Table A. Variable description and sources used for all analyses. Refer to S1 Data available on Scholars Portal for additional variable descriptions and source links.

Measure	Data source
**Country**	
Region defined by WHO	WHO
Population in 2020	UN
Population density (population/km^2)	World Bank
Participatory democracy index	V-dem
Proportion of population self-reporting trust in others	WVS
Emigrant population as percent of total population	UN
Passengers carried by air transport on air carriers registered in the country (domestic/international)	World Bank
Passengers carried by air transport per capita	World Bank
**Economy**	
Country income level	World Bank
GDP per capita	World Bank
Log of GDP	World Bank
GDP per capita (PPP int. $)	World Bank
Share of population living in extreme poverty	World Bank
**Gender**	
Political parity score	CFR
Current female head of state	CFR
Percentage of ministerial positions held by women	CFR
Percentage of seats held by women in national legislature	CFR
Percentage of women in parliament	World Bank
Percentage of elected seats held by women in local government bodies	CFR
**Health**	
Global Health Security Index	GHSI
Current health expenditure (% of GDP)	World Bank
Hospital beds per 1,000 people	OWID
**Domestic containment and closure indicator variables**	
School closure at some levels or categories (i.e.: high school/public schools) or at all levels	OxCGRT
Stay at home requirements with exceptions for daily exercise, grocery shopping, and essential trips	OxCGRT
Internal movement restrictions in-place	OxCGRT
Workplace closure for some sectors or categories of workers, and except for all-but-essential grocery stores or medical appointments	OxCGRT
Quarantine measures for incoming arrivals	OxCGRT
**Restriction**	
Cumulative domestic COVID-19 incidence at time of targeted restriction	Johns Hopkins University (JHU); own calculation
Cumulative domestic COVID-19 incidence at time of total restriction	JHU; own calculation
Cumulative domestic COVID-19 incidence at time of first restriction	JHU; own calculation
Timing of targeted restriction relative to other countries	OxCGRT; own calculation
Timing of total restriction relative to other countries	OxCGRT; own calculation

Timing of first restriction relative to other countries	OxCGRT; own calculation
Proportion of global cases targeted by first restriction	JHU; own calculation
Proportion of global population targeted by first restriction	UN; own calculation

Fig A. Global border closures changes in: COVID-19 R_t, incidence, testing capacity, and domestic containment and closure policies. Stacked area plot of the world's population living in countries with no border closures, targeted border closures, and total border closures. From left to right are the time-series trends of: i) R_t and changes in domestic containment and closure policies; and ii) Daily incidence and changes in domestic containment and closure policies.

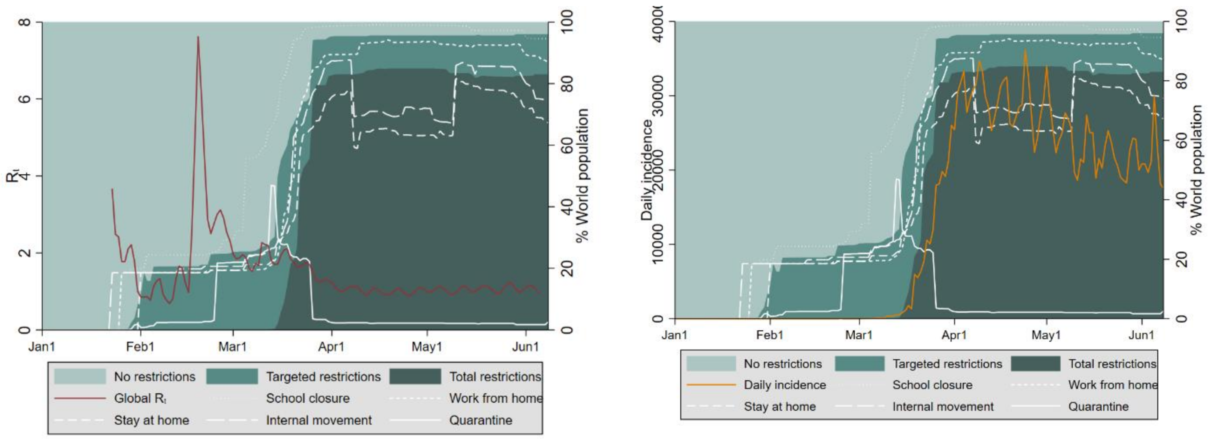

Fig B. Study flow diagram of the various stages of analyses. The flow of analyses through the different phases of our multimethod evaluation of the effectiveness of border closures mapping simultaneous and staged independent and interdependent analyses, and the logic of classification of treatment status by country.

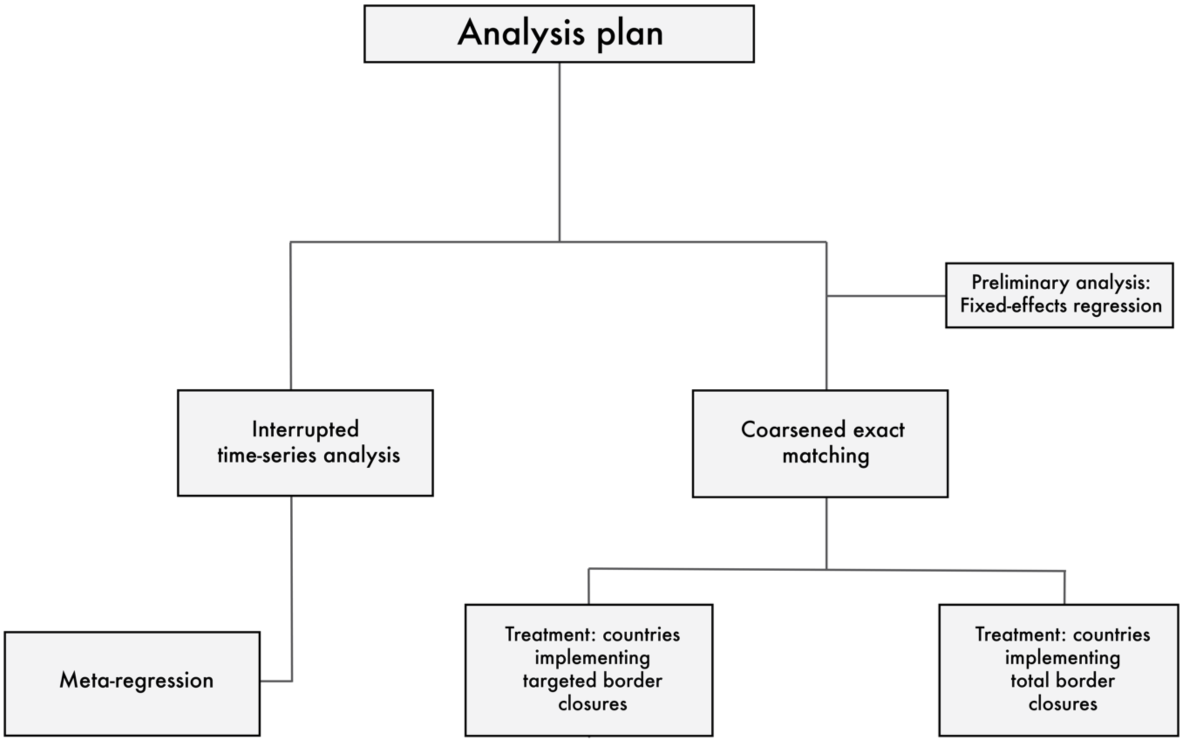

Fig C. Stratified interrupted time-series analyses of global R_t, excluding China. Time-series analyses of: i) Global border closure analysis of both targeted and total border closures for high-income countries; and ii) Global border closure analysis of both targeted and total border closures for low- and middle-income countries.

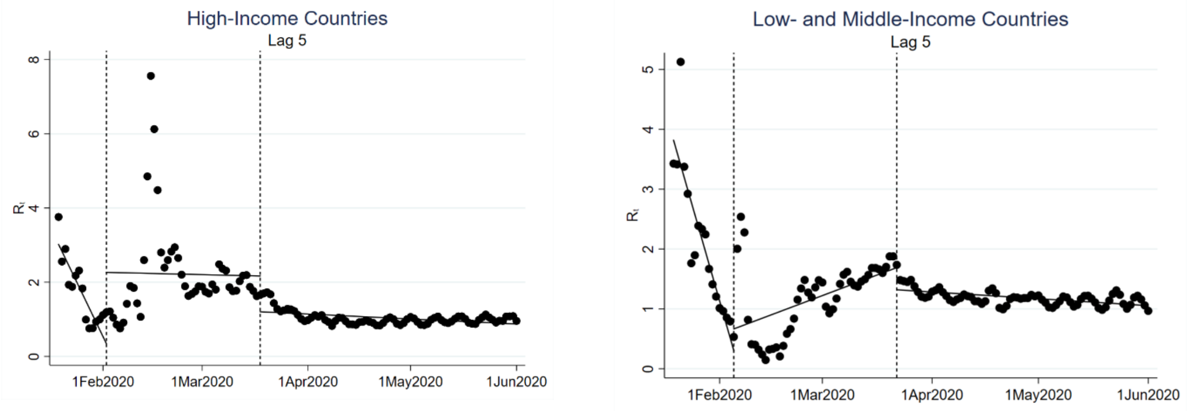

Table B. Global falsification tests with one intervention point for interrupted time-series results, excluding China. Results presented for 0 to 15-day lags.

	0-day lag	1-day lag	2-day lag	3-day lag	4-day lag	5-day lag	6-day lag	7-day lag
**Time**	-0.0136***	-0.0136***	-0.0136***	-0.0136***	-0.0135***	-0.0134***	-0.0134***	-0.0134***
**LCI**	-0.0199	-0.0198	-0.0197	-0.0196	-0.0194	-0.0193	-0.0192	-0.0191
**UCI**	-0.00734	-0.00744	-0.00750	-0.00751	-0.00750	-0.00751	-0.00755	-0.00763
**SE**	0.00318	0.00314	0.00310	0.00306	0.00302	0.00298	0.00295	0.00291
**Feb 5 level change**	0.0194	0.0424	0.0562	0.0605	0.0567	0.0602	0.0752	0.104
**LCI**	-0.146	-0.122	-0.109	-0.105	-0.108	-0.105	-0.0905	-0.0607
**UCI**	0.185	0.207	0.221	0.226	0.222	0.225	0.241	0.270
**SE**	0.0835	0.0833	0.0835	0.0838	0.0833	0.0833	0.0838	0.0835
**Feb 5 slope change**	0.0154***	0.0151***	0.0151***	0.0153***	0.0159***	0.0162***	0.0162***	0.0155***
**LCI**	0.00861	0.00837	0.00831	0.00855	0.00917	0.00951	0.00933	0.00863
**UCI**	0.0221	0.0219	0.0218	0.0221	0.0226	0.0230	0.0230	0.0223
**SE**	0.00342	0.00341	0.00342	0.00343	0.00340	0.00341	0.00345	0.00346
**Constant**	2.371***	2.371***	2.371***	2.368***	2.365***	2.363***	2.362***	2.363***
**LCI**	1.872	1.875	1.876	1.877	1.876	1.876	1.878	1.880
**UCI**	2.869	2.868	2.865	2.860	2.855	2.850	2.847	2.845
**SE**	0.252	0.251	0.250	0.249	0.247	0.246	0.245	0.244
**Observations**	135	135	135	135	135	135	135	135
	**8-day lag**	**9-day lag**	**10-day lag**	**11-day lag**	**12-day lag**	**13-day lag**	**14-day lag**	**15-day lag**
**Time**	-0.0134***	-0.0134***	-0.0133***	-0.0132***	-0.0131***	-0.0130***	-0.0129***	-0.0129***
**LCI**	-0.0191	-0.0190	-0.0188	-0.0187	-0.0185	-0.0183	-0.0182	-0.0181
**UCI**	-0.00770	-0.00775	-0.00774	-0.00770	-0.00765	-0.00764	-0.00766	-0.00767
**SE**	0.00287	0.00284	0.00280	0.00277	0.00274	0.00271	0.00267	0.00264
**Feb 5 level change**	0.136	0.163**	0.173**	0.169**	0.162*	0.171**	0.196**	0.221***
**LCI**	-0.0265	0.00110	0.00988	0.00508	-0.00256	0.00464	0.0290	0.0554
**UCI**	0.300	0.324	0.336	0.334	0.327	0.338	0.363	0.388
**SE**	0.0824	0.0817	0.0824	0.0831	0.0832	0.0843	0.0844	0.0840
**Feb 5 slope change**	0.0145***	0.0137***	0.0135***	0.0140***	0.0147***	0.0146***	0.0136***	0.0123***
**LCI**	0.00781	0.00706	0.00673	0.00705	0.00774	0.00732	0.00612	0.00482
**UCI**	0.0213	0.0203	0.0203	0.0209	0.0217	0.0219	0.0210	0.0197
**SE**	0.00340	0.00336	0.00343	0.00350	0.00353	0.00369	0.00377	0.00376
**Constant**	2.363***	2.362***	2.359***	2.355***	2.351***	2.348***	2.346***	2.345***
**LCI**	1.883	1.884	1.884	1.882	1.881	1.880	1.881	1.881
**UCI**	2.843	2.839	2.834	2.828	2.821	2.816	2.812	2.808
**SE**	0.243	0.241	0.240	0.239	0.238	0.237	0.235	0.234
**Observations**	135	135	135	135	135	135	135	135

*** $p < 0.01$, ** $p < 0.05$, * $p < 0.10$

Table C. Time-aligned analysis using unweighted average R_t. Results presented for countries with total border closures only, for 0 to 15-day lags.

	0-day lag	1-day lag	2-day lag	3-day lag	4-day lag	5-day lag	6-day lag	7-day lag
**Time**	0.0253***	0.0281***	0.0312***	0.0363***	0.0418***	0.0471***	0.0506***	0.0548***
**LCI**	0.0186	0.0216	0.0242	0.0278	0.0311	0.0356	0.0401	0.0463
**UCI**	0.0320	0.0345	0.0383	0.0448	0.0526	0.0586	0.0610	0.0632
**SE**	0.00332	0.00324	0.00352	0.00425	0.00537	0.00575	0.00522	0.00420
**Feb 5 level change**	0.852***	0.860***	0.843***	0.750***	0.604***	0.435**	0.317*	0.167
**LCI**	0.581	0.621	0.618	0.496	0.301	0.107	-0.0129	-0.165
**UCI**	1.122	1.099	1.068	1.004	0.906	0.762	0.647	0.500
**SE**	0.135	0.119	0.112	0.127	0.151	0.164	0.165	0.166
**Feb 5 slope change**	-0.0479***	-0.0567***	-0.0658***	-0.0751***	-0.0829***	-0.0884***	-0.0923***	-0.0955***
**LCI**	-0.0649	-0.0712	-0.0786	-0.0883	-0.0979	-0.104	-0.108	-0.111
**UCI**	-0.0310	-0.0423	-0.0529	-0.0619	-0.0679	-0.0725	-0.0766	-0.0801
**SE**	0.00847	0.00721	0.00642	0.00660	0.00747	0.00790	0.00781	0.00769
**Constant**	1.228***	1.214***	1.201***	1.167***	1.139***	1.115***	1.107***	1.089***
**LCI**	1.125	1.110	1.088	1.043	0.990	0.954	0.957	0.974
**UCI**	1.330	1.317	1.315	1.291	1.288	1.275	1.257	1.204
**SE**	0.0513	0.0517	0.0567	0.0621	0.0743	0.0801	0.0750	0.0575
**Observations**	61	61	61	61	61	61	61	61
	**8-day lag**	**9-day lag**	**10-day lag**	**11-day lag**	**12-day lag**	**13-day lag**	**14-day lag**	**15-day lag**
**Time**	0.0573***	0.0566***	0.0543***	0.0503***	0.0479***	0.0469***	0.0489***	0.0483***
**LCI**	0.0501	0.0488	0.0447	0.0382	0.0360	0.0359	0.0383	0.0387
**UCI**	0.0644	0.0645	0.0640	0.0623	0.0599	0.0578	0.0595	0.0580
**SE**	0.00359	0.00391	0.00480	0.00602	0.00598	0.00549	0.00529	0.00480
**Feb 5 level change**	0.0365	-0.00487	-0.00196	0.0788	0.0949	0.0500	-0.114	-0.212
**LCI**	-0.305	-0.366	-0.382	-0.304	-0.274	-0.310	-0.486	-0.591
**UCI**	0.378	0.356	0.378	0.461	0.464	0.410	0.257	0.167
**SE**	0.171	0.180	0.190	0.191	0.184	0.180	0.185	0.189
**Feb 5 slope change**	-0.0970***	-0.0968***	-0.0953***	-0.0953***	-0.0952***	-0.0949***	-0.0945***	-0.0923***
**LCI**	-0.113	-0.114	-0.113	-0.113	-0.112	-0.111	-0.111	-0.109
**UCI**	-0.0813	-0.0799	-0.0772	-0.0772	-0.0780	-0.0784	-0.0778	-0.0753
**SE**	0.00786	0.00842	0.00904	0.00904	0.00859	0.00823	0.00833	0.00848
**Constant**	1.101***	1.149***	1.216***	1.303***	1.370***	1.426***	1.447***	1.499***
**LCI**	1.001	1.053	1.115	1.188	1.260	1.325	1.351	1.408
**UCI**	1.201	1.244	1.317	1.418	1.480	1.527	1.543	1.590
**SE**	0.0499	0.0478	0.0506	0.0575	0.0549	0.0504	0.0480	0.0455
**Observations**	61	61	61	61	61	61	61	61

*** $p < 0.01$, ** $p < 0.05$, * $p < 0.10$

Table D. Time-aligned analysis using population-weighted average R_t. Results presented for countries with total border closures only, for 0 to 15-day lags.

	**0-day lag**	**1-day lag**	**2-day lag**	**3-day lag**	**4-day lag**	**5-day lag**	**6-day lag**	**7-day lag**
**Time**	0.0225***	0.0250***	0.0279***	0.0338***	0.0402***	0.0470***	0.0522***	0.0556***
**LCI**	0.0147	0.0177	0.0205	0.0239	0.0261	0.0318	0.0383	0.0438
**UCI**	0.0304	0.0322	0.0353	0.0438	0.0544	0.0621	0.0660	0.0674
**SE**	0.00392	0.00364	0.00369	0.00496	0.00708	0.00756	0.00690	0.00589
**Feb 5 level change**	0.914***	0.952***	0.963***	0.860***	0.682***	0.490**	0.343	0.244
**LCI**	0.565	0.645	0.689	0.543	0.290	0.0683	-0.0840	-0.182
**UCI**	1.262	1.259	1.238	1.177	1.073	0.912	0.770	0.670
**SE**	0.174	0.153	0.137	0.158	0.195	0.211	0.213	0.213
**Feb 5 slope change**	-0.0427***	-0.0515***	-0.0610***	-0.0711***	-0.0795***	-0.0868***	-0.0929***	-0.0973***
**LCI**	-0.0615	-0.0675	-0.0746	-0.0863	-0.0985	-0.107	-0.113	-0.117
**UCI**	-0.0239	-0.0356	-0.0474	-0.0559	-0.0604	-0.0665	-0.0727	-0.0775
**SE**	0.00939	0.00795	0.00680	0.00761	0.00952	0.0102	0.0101	0.00987
**Constant**	1.225***	1.208***	1.191***	1.146***	1.114***	1.069***	1.038***	1.025***
**LCI**	1.101	1.087	1.064	0.999	0.921	0.863	0.848	0.869
**UCI**	1.350	1.330	1.319	1.294	1.306	1.274	1.228	1.182
**SE**	0.0621	0.0607	0.0636	0.0736	0.0962	0.103	0.0949	0.0781
**Observations**	61	61	61	61	61	61	61	61
	**8-day lag**	**9-day lag**	**10-day lag**	**11-day lag**	**12-day lag**	**13-day lag**	**14-day lag**	**15-day lag**
**Time**	0.0595***	0.0585***	0.0564***	0.0528***	0.0513***	0.0512***	0.0555***	0.0566***
**LCI**	0.0489	0.0470	0.0437	0.0391	0.0386	0.0396	0.0418	0.0438
**UCI**	0.0700	0.0700	0.0692	0.0666	0.0640	0.0628	0.0693	0.0693
**SE**	0.00527	0.00574	0.00635	0.00688	0.00634	0.00578	0.00687	0.00636
**Feb 5 level change**	0.0700	0.0663	0.0884	0.168	0.146	0.0621	-0.206	-0.374*
**LCI**	-0.365	-0.383	-0.365	-0.269	-0.274	-0.354	-0.639	-0.802
**UCI**	0.505	0.515	0.542	0.604	0.566	0.478	0.226	0.0546
**SE**	0.217	0.224	0.226	0.218	0.210	0.208	0.216	0.214
**Feb 5 slope change**	-0.0990***	-0.0991***	-0.0984***	-0.0998***	-0.100***	-0.101***	-0.101***	-0.0995***
**LCI**	-0.119	-0.121	-0.120	-0.121	-0.120	-0.121	-0.121	-0.119
**UCI**	-0.0785	-0.0776	-0.0764	-0.0787	-0.0803	-0.0813	-0.0810	-0.0796
**SE**	0.0102	0.0107	0.0110	0.0105	0.00999	0.00981	0.0101	0.00992
**Constant**	1.020***	1.065***	1.122***	1.206***	1.273***	1.328***	1.338***	1.380***
**LCI**	0.879	0.937	1.003	1.081	1.159	1.220	1.203	1.250
**UCI**	1.162	1.192	1.241	1.330	1.388	1.435	1.472	1.510
**SE**	0.0706	0.0637	0.0593	0.0621	0.0572	0.0538	0.0671	0.0650
**Observations**	61	61	61	61	61	61	61	61

*** p < 0.01, ** p < 0.05, * p < 0.10

Table E. Interrupted time-series results of the global time-varying effective reproductive number (R_t), excluding extreme values above R_t of 4 (four data points dropped). The first global intervention is of targeted border closures on February 5, 2020, then followed by the second global intervention of total border closures on March 19, 2020. Results presented for 0 to 12-day lags.

	0-day lag	1-day lag	2-day lag	3-day lag	4-day lag	5-day lag	6-day lag	7-day lag
**Time**	-0.200***	-0.177***	-0.154***	-0.134***	-0.126***	-0.121***	-0.116***	-0.110***
**LCI**	-0.258	-0.240	-0.218	-0.196	-0.179	-0.167	-0.157	-0.148
**UCI**	-0.141	-0.114	-0.0899	-0.0724	-0.0730	-0.0742	-0.0751	-0.0714
**SE**	0.0296	0.0319	0.0325	0.0313	0.0269	0.0234	0.0208	0.0193
**Feb 5 level change**	1.052***	1.094***	1.089***	1.082***	1.230***	1.417***	1.626***	1.798***
**LCI**	0.573	0.502	0.419	0.370	0.549	0.765	1.012	1.213
**UCI**	1.532	1.685	1.759	1.793	1.910	2.068	2.240	2.383
**SE**	0.242	0.299	0.339	0.360	0.344	0.329	0.310	0.296
**Feb 5 slope change**	0.221***	0.196***	0.171***	0.148***	0.136***	0.126***	0.116***	0.104***
**LCI**	0.162	0.132	0.105	0.0849	0.0806	0.0765	0.0717	0.0626
**UCI**	0.280	0.260	0.236	0.212	0.191	0.175	0.160	0.145
**SE**	0.0300	0.0324	0.0331	0.0321	0.0280	0.0248	0.0222	0.0207
**Mar 19 level change**	-0.992***	-0.990***	-0.980***	-0.954***	-0.908***	-0.859***	-0.807***	-0.749***
**LCI**	-1.258	-1.248	-1.238	-1.222	-1.178	-1.121	-1.048	-0.965
**UCI**	-0.726	-0.732	-0.722	-0.686	-0.639	-0.598	-0.567	-0.532
**SE**	0.135	0.130	0.130	0.136	0.136	0.132	0.122	0.110
**Mar 19 slope change**	-0.0264***	-0.0234***	-0.0206***	-0.0176**	-0.0129	-0.00747	-0.00139	0.00482
**LCI**	-0.0371	-0.0351	-0.0335	-0.0320	-0.0283	-0.0234	-0.0170	-0.0100
**UCI**	-0.0156	-0.0118	-0.00779	-0.00324	0.00256	0.00850	0.0142	0.0196
**SE**	0.00545	0.00589	0.00649	0.00725	0.00781	0.00807	0.00788	0.00749
**Constant**	2.931***	2.841***	2.742***	2.649***	2.608***	2.579***	2.554***	2.515***
**LCI**	2.440	2.331	2.215	2.111	2.089	2.076	2.064	2.028
**UCI**	3.422	3.350	3.269	3.187	3.128	3.082	3.044	3.001
**SE**	0.248	0.258	0.266	0.272	0.262	0.254	0.247	0.246
**Observations**	131	131	131	131	131	131	131	131

	8-day lag	9-day lag	10-day lag	11-day lag	12-day lag
**Time**	-0.0968***	-0.0819***	-0.0703***	-0.0645***	-0.0616***
**LCI**	-0.138	-0.126	-0.114	-0.104	-0.0975
**UCI**	-0.0558	-0.0375	-0.0267	-0.0248	-0.0258
**SE**	0.0207	0.0225	0.0221	0.0201	0.0181
**Feb 5 level change**	1.829***	1.780***	1.775***	1.900***	2.107***
**LCI**	1.184	1.050	1.020	1.209	1.594
**UCI**	2.473	2.511	2.530	2.590	2.621
**SE**	0.326	0.369	0.382	0.349	0.260
**Feb 5 slope change**	0.0861***	0.0671***	0.0509**	0.0386*	0.0277
**LCI**	0.0423	0.0196	0.00377	-0.00431	-0.00887
**UCI**	0.130	0.115	0.0980	0.0814	0.0644
**SE**	0.0221	0.0240	0.0238	0.0217	0.0185
**Mar 19 level change**	-0.679***	-0.601***	-0.510***	-0.394***	-0.262***
**LCI**	-0.902	-0.848	-0.775	-0.641	-0.392
**UCI**	-0.456	-0.354	-0.244	-0.148	-0.131
**SE**	0.113	0.125	0.134	0.124	0.0659
**Mar 19 slope change**	0.00992	0.0142*	0.0191**	0.0259***	0.0342***
**Feb 5 level change**	-0.00553	-0.00257	0.00133	0.00964	0.0265
**LCI**	0.0254	0.0310	0.0369	0.0421	0.0418
**SE**	0.00781	0.00849	0.00898	0.00820	0.00387
**Constant**	2.433***	2.334***	2.253***	2.210***	2.188***
**LCI**	1.921	1.787	1.692	1.659	1.652
**UCI**	2.946	2.880	2.813	2.761	2.724
**SE**	0.259	0.276	0.283	0.278	0.271
**Observations**	131	131	131	131	131

*** $p < 0.01$, ** $p < 0.05$, * $p < 0.10$

Table F. Interrupted time-series results of the global time-varying effective reproductive number (R_t), excluding extreme values above R_t of 4 (four data points dropped). The global intervention is of total border closures on March 19, 2020. Results presented for 0 to 15-day lags.

	0-day lag	1-day lag	2-day lag	3-day lag	4-day lag	5-day lag	6-day lag	7-day lag
**Time**	0.00919	0.00876	0.00820	0.00747	0.00683	0.00632	0.00592	0.00547
**LCI**	-0.00205	-0.00208	-0.00228	-0.00269	-0.00302	-0.00322	-0.00332	-0.00349
**UCI**	0.0204	0.0196	0.0187	0.0176	0.0167	0.0159	0.0152	0.0144
**SE**	0.00568	0.00548	0.00530	0.00514	0.00498	0.00482	0.00467	0.00453
**Feb 5 level change**	-0.768***	-0.793***	-0.808***	-0.810***	-0.817***	-0.832***	-0.855***	-0.875***
**LCI**	-1.058	-1.073	-1.080	-1.078	-1.080	-1.087	-1.098	-1.106
**UCI**	-0.478	-0.513	-0.536	-0.541	-0.554	-0.577	-0.612	-0.643
**SE**	0.146	0.141	0.138	0.136	0.133	0.129	0.123	0.117
**Feb 5 slope change**	-0.0142**	-0.0132**	-0.0122**	-0.0110**	-0.00993*	-0.00888*	-0.00783	-0.00673
**LCI**	-0.0256	-0.0243	-0.0229	-0.0214	-0.0200	-0.0186	-0.0172	-0.0158
**UCI**	-0.00268	-0.00213	-0.00145	-0.000626	0.000156	0.000890	0.00160	0.00236
**SE**	0.00580	0.00559	0.00541	0.00526	0.00510	0.00494	0.00476	0.00459
**Constant**	1.570***	1.577***	1.588***	1.601***	1.613***	1.623***	1.630***	1.639***
**LCI**	1.128	1.141	1.157	1.176	1.193	1.208	1.220	1.233
**UCI**	2.012	2.014	2.018	2.026	2.033	2.038	2.041	2.045
**SE**	0.223	0.221	0.218	0.215	0.212	0.210	0.207	0.205
**Observations**	131	131	131	131	131	131	131	131
	**8-day lag**	**9-day lag**	**10-day lag**	**11-day lag**	**12-day lag**	**13-day lag**	**14-day lag**	**15-day lag**
**Time**	0.00473	0.00382	0.00291	0.00211	0.00142	0.000756	8.62e-05	-0.000646
**LCI**	-0.00401	-0.00476	-0.00551	-0.00613	-0.00662	-0.00709	-0.00758	-0.00816
**UCI**	0.0135	0.0124	0.0113	0.0103	0.00946	0.00860	0.00775	0.00686
**SE**	0.00442	0.00434	0.00426	0.00416	0.00406	0.00397	0.00387	0.00380
**Feb 5 level change**	-0.869***	-0.846***	-0.820***	-0.798***	-0.784***	-0.769***	-0.750***	-0.721***
**LCI**	-1.098	-1.078	-1.053	-1.032	-1.014	-0.995	-0.974	-0.945
**UCI**	-0.641	-0.615	-0.586	-0.565	-0.554	-0.542	-0.526	-0.498
**SE**	0.115	0.117	0.118	0.118	0.116	0.114	0.113	0.113
**Feb 5 slope change**	-0.00556	-0.00438	-0.00325	-0.00217	-0.00114	-0.000127	0.000848	0.00176
**LCI**	-0.0144	-0.0131	-0.0118	-0.0105	-0.00928	-0.00806	-0.00690	-0.00583
**UCI**	0.00329	0.00431	0.00529	0.00618	0.00701	0.00781	0.00859	0.00935
**SE**	0.00447	0.00439	0.00431	0.00422	0.00411	0.00401	0.00391	0.00384
**Constant**	1.654***	1.673***	1.692***	1.709***	1.724***	1.738***	1.753***	1.770***
**LCI**	1.252	1.275	1.298	1.319	1.337	1.356	1.374	1.394
**UCI**	2.056	2.071	2.086	2.099	2.110	2.121	2.133	2.146
**SE**	0.203	0.201	0.199	0.197	0.195	0.193	0.192	0.190
**Observations**	131	131	131	131	131	131	131	131

*** $p < 0.01$, ** $p < 0.05$, * $p < 0.10$

Table G. Interrupted time-series results censoring for 45 days only, before and after the two intervention points. Results are presented for 0 to 15-day lags.

	0-day lag	1-day lag	2-day lag	3-day lag	4-day lag	5-day lag	6-day lag	7-day lag
**Time**	-0.200***	-0.177***	-0.154***	-0.134***	-0.126***	-0.121***	-0.116***	-0.110***
**LCI**	-0.259	-0.241	-0.219	-0.197	-0.180	-0.167	-0.158	-0.148
**UCI**	-0.140	-0.113	-0.0894	-0.0719	-0.0725	-0.0739	-0.0748	-0.0712
**SE**	0.0298	0.0321	0.0327	0.0314	0.0270	0.0236	0.0209	0.0194
**Feb 5 level change**	1.776***	1.866***	1.911***	1.951***	2.139***	2.359***	2.593***	2.786***
**LCI**	0.820	0.812	0.775	0.759	0.946	1.172	1.426	1.639
**UCI**	2.732	2.920	3.046	3.144	3.332	3.545	3.760	3.933
**SE**	0.482	0.531	0.572	0.601	0.601	0.598	0.588	0.578
**Feb 5 slope change**	0.205***	0.177***	0.150***	0.125***	0.110***	0.0981***	0.0866***	0.0731***
**LCI**	0.140	0.108	0.0785	0.0547	0.0470	0.0398	0.0322	0.0210
**UCI**	0.269	0.247	0.221	0.195	0.174	0.156	0.141	0.125
**SE**	0.0324	0.0349	0.0359	0.0353	0.0319	0.0294	0.0274	0.0263
**Mar 19 level change**	-0.809***	-0.777***	-0.736***	-0.673***	-0.591***	-0.507**	-0.426*	-0.343
**LCI**	-1.178	-1.143	-1.117	-1.081	-1.018	-0.947	-0.871	-0.791
**UCI**	-0.439	-0.410	-0.355	-0.265	-0.164	-0.0675	0.0181	0.105
**SE**	0.186	0.185	0.192	0.206	0.215	0.222	0.224	0.226
**Mar 19 slope change**	-0.0208	-0.0143	-0.00786	-0.00159	0.00598	0.0139	0.0224	0.0309*
**LCI**	-0.0463	-0.0418	-0.0377	-0.0339	-0.0280	-0.0211	-0.0129	-0.00428
**UCI**	0.00463	0.0133	0.0220	0.0307	0.0400	0.0490	0.0577	0.0661
**SE**	0.0128	0.0139	0.0151	0.0163	0.0171	0.0177	0.0178	0.0177
**Constant**	2.931***	2.841***	2.742***	2.649***	2.608***	2.579***	2.554***	2.515***
**LCI**	2.435	2.326	2.210	2.106	2.085	2.072	2.061	2.025
**UCI**	3.427	3.355	3.274	3.192	3.132	3.086	3.047	3.005
**SE**	0.250	0.259	0.268	0.274	0.264	0.256	0.249	0.247
**Observations**	100	100	100	100	100	100	100	100
	**8-day lag**	**9-day lag**	**10-day lag**	**11-day lag**	**12-day lag**	**13-day lag**	**14-day lag**	**15-day lag**
**Time**	-0.0968***	-0.0819***	-0.0703***	-0.0645***	-0.0616***	-0.0457**	-0.0133	0.0324
**LCI**	-0.138	-0.127	-0.114	-0.104	-0.0976	-0.0888	-0.0825	-0.0678
**UCI**	-0.0556	-0.0372	-0.0264	-0.0246	-0.0256	-0.00256	0.0559	0.133
**SE**	0.0208	0.0226	0.0221	0.0201	0.0182	0.0218	0.0349	0.0506
**Feb 5 level change**	2.840***	2.817***	2.828***	2.951***	3.135***	2.939***	2.180***	0.855
**LCI**	1.666	1.599	1.612	1.803	2.113	1.839	0.675	-1.096
**UCI**	4.013	4.034	4.045	4.098	4.157	4.039	3.685	2.806
**SE**	0.592	0.614	0.613	0.579	0.515	0.555	0.759	0.984
**Feb 5 slope change**	0.0540*	0.0336	0.0161	0.00324	-0.00735	-0.0260	-0.0530	-0.0842
**LCI**	-0.000480	-0.0241	-0.0411	-0.0499	-0.0550	-0.0792	-0.129	-0.187
**UCI**	0.109	0.0912	0.0732	0.0564	0.0403	0.0272	0.0234	0.0183
**SE**	0.0275	0.0291	0.0288	0.0268	0.0240	0.0268	0.0386	0.0518
**Mar 19 level change**	-0.246	-0.140	-0.0230	0.110	0.251	0.321	0.269	0.0874
**LCI**	-0.714	-0.634	-0.535	-0.394	-0.214	-0.153	-0.224	-0.245
**UCI**	0.221	0.354	0.489	0.615	0.716	0.794	0.762	0.420
**SE**	0.236	0.249	0.258	0.255	0.235	0.239	0.249	0.168
**Mar 19 slope change**	0.0382**	0.0447**	0.0514***	0.0591***	0.0675***	0.0708***	0.0661***	0.0522***
**LCI**	0.00253	0.00824	0.0147	0.0240	0.0362	0.0396	0.0335	0.0301
**UCI**	0.0740	0.0812	0.0881	0.0943	0.0987	0.102	0.0986	0.0743
**SE**	0.0180	0.0184	0.0185	0.0177	0.0158	0.0157	0.0164	0.0111
**Constant**	2.433***	2.334***	2.253***	2.210***	2.188***	2.061***	1.791***	1.395**
**LCI**	1.918	1.784	1.689	1.656	1.649	1.459	0.982	0.292
**UCI**	2.949	2.884	2.816	2.764	2.727	2.662	2.599	2.497
**SE**	0.260	0.277	0.284	0.280	0.272	0.303	0.408	0.556
**Observations**	100	100	100	100	100	100	100	100

*** $p < 0.01$, ** $p < 0.05$, * $p < 0.10$

Table H. Interrupted time-series results censoring for 45 days after the global total border closure intervention. Results are presented for 0 to 15-day lags.

	0-day lag	1-day lag	2-day lag	3-day lag	4-day lag	5-day lag	6-day lag	7-day lag
**Time**	-0.200***	-0.177***	-0.154***	-0.134***	-0.126***	-0.121***	-0.116***	-0.110***
**LCI**	-0.259	-0.241	-0.219	-0.197	-0.180	-0.168	-0.158	-0.148
**UCI**	-0.140	-0.113	-0.0892	-0.0718	-0.0724	-0.0737	-0.0746	-0.0710
**SE**	0.0298	0.0321	0.0327	0.0315	0.0271	0.0236	0.0210	0.0195
**Feb 5 level change**	1.776***	1.866***	1.911***	1.951***	2.139***	2.359***	2.593***	2.786***
**LCI**	0.819	0.811	0.773	0.756	0.943	1.169	1.422	1.635
**UCI**	2.733	2.922	3.048	3.147	3.335	3.548	3.764	3.937
**SE**	0.482	0.531	0.573	0.602	0.602	0.599	0.590	0.580
**Feb 5 slope change**	0.205***	0.177***	0.150***	0.125***	0.110***	0.0981***	0.0866***	0.0731***
**LCI**	0.140	0.108	0.0784	0.0546	0.0469	0.0396	0.0321	0.0208
**UCI**	0.269	0.247	0.221	0.195	0.174	0.156	0.141	0.125
**SE**	0.0324	0.0350	0.0359	0.0354	0.0320	0.0294	0.0275	0.0263
**Mar 19 level change**	-0.791***	-0.749***	-0.697***	-0.626***	-0.541**	-0.459**	-0.384*	-0.306
**LCI**	-1.161	-1.115	-1.077	-1.034	-0.969	-0.901	-0.831	-0.756
**UCI**	-0.422	-0.382	-0.317	-0.218	-0.112	-0.0166	0.0638	0.144
**SE**	0.186	0.185	0.192	0.206	0.216	0.223	0.225	0.227
**Mar 19 slope change**	-0.0220*	-0.0162	-0.0106	-0.00493	0.00232	0.0104	0.0192	0.0280
**LCI**	-0.0476	-0.0438	-0.0406	-0.0374	-0.0319	-0.0249	-0.0164	-0.00737
**UCI**	0.00350	0.0114	0.0194	0.0275	0.0365	0.0456	0.0547	0.0635
**SE**	0.0129	0.0139	0.0151	0.0163	0.0172	0.0178	0.0179	0.0178
**Constant**	2.931***	2.841***	2.742***	2.649***	2.608***	2.579***	2.554***	2.515***
**LCI**	2.435	2.325	2.209	2.105	2.083	2.070	2.059	2.023
**UCI**	3.428	3.356	3.275	3.193	3.134	3.088	3.049	3.007
**SE**	0.250	0.260	0.268	0.274	0.264	0.256	0.249	0.248
**Observations**	100	100	100	100	100	100	100	100

	8-day lag	9-day lag	10-day lag	11-day lag	12-day lag	13-day lag	14-day lag	15-day lag
**Time**	-0.0968***	-0.0819***	-0.0703***	-0.0645***	-0.0616***	-0.0457**	-0.0133	0.0324
**LCI**	-0.138	-0.127	-0.114	-0.105	-0.0978	-0.0891	-0.0829	-0.0684
**UCI**	-0.0554	-0.0370	-0.0262	-0.0244	-0.0254	-0.00230	0.0563	0.133
**SE**	0.0209	0.0226	0.0222	0.0202	0.0182	0.0219	0.0351	0.0508
**Feb 5 level change**	2.840***	2.817***	2.828***	2.951***	3.135***	2.939***	2.180***	0.855
**LCI**	1.661	1.594	1.606	1.797	2.107	1.832	0.666	-1.109
**UCI**	4.018	4.039	4.051	4.104	4.162	4.046	3.695	2.819
**SE**	0.594	0.616	0.616	0.581	0.517	0.557	0.763	0.989
**Feb 5 slope change**	0.0540*	0.0336	0.0161	0.00324	-0.00735	-0.0260	-0.0530	-0.0842
**LCI**	-0.000707	-0.0244	-0.0414	-0.0502	-0.0553	-0.0796	-0.130	-0.187
**UCI**	0.109	0.0915	0.0735	0.0567	0.0406	0.0275	0.0239	0.0190
**SE**	0.0276	0.0292	0.0289	0.0269	0.0241	0.0270	0.0387	0.0520
**Mar 19 level change**	-0.210	-0.0990	0.0230	0.156	0.289	0.351	0.295	0.113
**LCI**	-0.679	-0.595	-0.492	-0.353	-0.179	-0.126	-0.202	-0.222
**UCI**	0.260	0.397	0.538	0.664	0.758	0.829	0.791	0.449
**SE**	0.237	0.250	0.259	0.256	0.236	0.240	0.250	0.169
**Mar 19 slope change**	0.0354*	0.0415**	0.0478**	0.0555***	0.0643***	0.0681***	0.0638***	0.0499***
**LCI**	-0.000567	0.00476	0.0108	0.0200	0.0326	0.0366	0.0309	0.0275
**UCI**	0.0713	0.0782	0.0848	0.0909	0.0959	0.0997	0.0967	0.0723
**SE**	0.0181	0.0185	0.0186	0.0179	0.0159	0.0159	0.0166	0.0113
**Constant**	2.433***	2.334***	2.253***	2.210***	2.188***	2.061***	1.791***	1.395**
**LCI**	1.915	1.781	1.686	1.653	1.646	1.456	0.977	0.285
**UCI**	2.951	2.886	2.819	2.767	2.730	2.665	2.605	2.504
**SE**	0.261	0.278	0.285	0.281	0.273	0.305	0.410	0.559
**Observations**	100	100	100	100	100	100	100	100

*** $p < 0.01$, ** $p < 0.05$, * $p < 0.10$

Table I. Interrupted time-series analyses. Results of countries with quarantine measures, with two global intervention points. Results are presented 0 to 15-day lags.

	0-day lag	1-day lag	2-day lag	3-day lag	4-day lag	5-day lag	6-day lag	7-day lag
**Time**	0.00574	0.00557	0.00491	0.00412	0.00326	0.00257	0.00207	0.00156
**LCI**	-0.0110	-0.0105	-0.0106	-0.0109	-0.0112	-0.0114	-0.0114	-0.0115
**UCI**	0.0225	0.0217	0.0204	0.0191	0.0177	0.0166	0.0156	0.0146
**SE**	0.00846	0.00814	0.00784	0.00757	0.00732	0.00707	0.00683	0.00661
**Feb 5 level change**	-0.801***	-0.841***	-0.846***	-0.838***	-0.822***	-0.817***	-0.826***	-0.833***
**LCI**	-1.286	-1.306	-1.298	-1.280	-1.256	-1.240	-1.235	-1.229
**UCI**	-0.316	-0.376	-0.394	-0.396	-0.389	-0.395	-0.416	-0.436
**SE**	0.245	0.235	0.229	0.223	0.219	0.214	0.207	0.200
**Feb 5 slope change**	-0.0117	-0.0109	-0.00978	-0.00865	-0.00753	-0.00648	-0.00546	-0.00444
**LCI**	-0.0286	-0.0271	-0.0254	-0.0238	-0.0221	-0.0206	-0.0191	-0.0176
**UCI**	0.00515	0.00535	0.00585	0.00645	0.00708	0.00765	0.00818	0.00874
**SE**	0.00853	0.00820	0.00790	0.00763	0.00739	0.00714	0.00690	0.00666
**Constant**	1.908***	1.911***	1.924***	1.941***	1.958***	1.973***	1.984***	1.995***
**LCI**	1.249	1.261	1.284	1.310	1.337	1.360	1.379	1.398
**UCI**	2.567	2.561	2.565	2.571	2.580	2.586	2.588	2.592
**SE**	0.333	0.329	0.324	0.319	0.314	0.310	0.306	0.302
**Observations**	139	139	139	139	139	139	139	139
	**8-day lag**	**9-day lag**	**10-day lag**	**11-day lag**	**12-day lag**	**13-day lag**	**14-day lag**	**15-day lag**
**Time**	0.000896	-1.80e-05	-0.000905	-0.00159	-0.00209	-0.00256	-0.00314	-0.00372
**LCI**	-0.0118	-0.0123	-0.0129	-0.0132	-0.0133	-0.0135	-0.0138	-0.0141
**UCI**	0.0136	0.0123	0.0111	0.0100	0.00917	0.00836	0.00748	0.00661
**SE**	0.00640	0.00622	0.00605	0.00587	0.00569	0.00552	0.00537	0.00522
**Feb 5 level change**	-0.824***	-0.790***	-0.754***	-0.732***	-0.726***	-0.721***	-0.701***	-0.679***
**LCI**	-1.211	-1.172	-1.131	-1.102	-1.086	-1.071	-1.044	-1.015
**UCI**	-0.438	-0.408	-0.376	-0.362	-0.366	-0.371	-0.359	-0.344
**SE**	0.196	0.193	0.191	0.187	0.182	0.177	0.173	0.170
**Feb 5 slope change**	-0.00340	-0.00237	-0.00141	-0.000506	0.000393	0.00129	0.00213	0.00293
**LCI**	-0.0162	-0.0148	-0.0135	-0.0123	-0.0110	-0.00977	-0.00863	-0.00755
**UCI**	0.00937	0.0100	0.0107	0.0112	0.0118	0.0123	0.0129	0.0134
**SE**	0.00645	0.00628	0.00612	0.00594	0.00576	0.00559	0.00544	0.00530
**Constant**	2.009***	2.030***	2.050***	2.065***	2.077***	2.088***	2.102***	2.116***
**LCI**	1.420	1.448	1.475	1.498	1.516	1.533	1.553	1.573
**UCI**	2.598	2.611	2.624	2.633	2.638	2.643	2.651	2.659
**SE**	0.298	0.294	0.290	0.287	0.284	0.281	0.277	0.275
**Observations**	139	139	139	139	139	139	139	139

*** $p < 0.01$, ** $p < 0.05$, * $p < 0.10$

Table J. Interrupted time-series results. Analyses of countries without quarantine measures, with two global intervention points. Results are presented 0 to 15-day lags.

	0-day lag	1-day lag	2-day lag	3-day lag	4-day lag	5-day lag	6-day lag	7-day lag
**Time**	-0.00571	-0.00667	-0.00709	-0.00723	-0.00731	-0.00780	-0.00834	-0.00902
**LCI**	-0.0232	-0.0234	-0.0232	-0.0227	-0.0222	-0.0222	-0.0223	-0.0226
**UCI**	0.0118	0.0101	0.00897	0.00824	0.00763	0.00664	0.00564	0.00456
**SE**	0.00886	0.00847	0.00813	0.00782	0.00755	0.00730	0.00707	0.00686
**Feb 5 level change**	-0.783**	-0.759***	-0.769***	-0.797***	-0.831***	-0.835***	-0.834***	-0.821***
**LCI**	-1.379	-1.330	-1.313	-1.315	-1.323	-1.305	-1.283	-1.253
**UCI**	-0.187	-0.189	-0.224	-0.279	-0.339	-0.365	-0.384	-0.389
**SE**	0.301	0.289	0.275	0.262	0.249	0.238	0.227	0.218
**Feb 5 slope change**	-0.00157	-0.000381	0.000521	0.00131	0.00212	0.00313	0.00417	0.00524
**LCI**	-0.0193	-0.0174	-0.0158	-0.0144	-0.0130	-0.0115	-0.01000	-0.00853
**UCI**	0.0161	0.0166	0.0168	0.0170	0.0172	0.0177	0.0183	0.0190
**SE**	0.00895	0.00858	0.00824	0.00793	0.00765	0.00739	0.00716	0.00696
**Constant**	2.540***	2.558***	2.566***	2.568***	2.570***	2.579***	2.590***	2.604***
**LCI**	1.831	1.854	1.865	1.870	1.874	1.886	1.900	1.916
**UCI**	3.249	3.262	3.266	3.266	3.265	3.272	3.280	3.291
**SE**	0.358	0.356	0.354	0.353	0.352	0.350	0.349	0.348
**Observations**	139	139	139	139	139	139	139	139

	8-day lag	9-day lag	10-day lag	11-day lag	12-day lag	13-day lag	14-day lag	15-day lag
**Time**	-0.00957	-0.00999	-0.0103	-0.0106*	-0.0112*	-0.0120**	-0.0127**	-0.0133**
**LCI**	-0.0228	-0.0228	-0.0228	-0.0229	-0.0232	-0.0237	-0.0242	-0.0247
**UCI**	0.00363	0.00286	0.00223	0.00160	0.000742	-0.000214	-0.00115	-0.00199
**SE**	0.00667	0.00650	0.00634	0.00619	0.00606	0.00594	0.00584	0.00573
**Feb 5 level change**	-0.816***	-0.818***	-0.830***	-0.838***	-0.822***	-0.791***	-0.756***	-0.726***
**LCI**	-1.230	-1.215	-1.207	-1.196	-1.167	-1.126	-1.083	-1.044
**UCI**	-0.401	-0.422	-0.452	-0.479	-0.476	-0.455	-0.429	-0.409
**SE**	0.210	0.200	0.191	0.181	0.175	0.170	0.165	0.161
**Feb 5 slope change**	0.00627	0.00726	0.00824	0.00925	0.0103*	0.0113*	0.0123**	0.0132**
**LCI**	-0.00712	-0.00577	-0.00443	-0.00308	-0.00176	-0.000510	0.000667	0.00180
**UCI**	0.0197	0.0203	0.0209	0.0216	0.0224	0.0231	0.0239	0.0246
**SE**	0.00677	0.00659	0.00641	0.00623	0.00610	0.00598	0.00588	0.00577
**Constant**	2.615***	2.624***	2.631***	2.638***	2.651***	2.668***	2.684***	2.699***
**LCI**	1.930	1.941	1.950	1.960	1.975	1.994	2.012	2.029
**UCI**	3.300	3.307	3.311	3.317	3.327	3.342	3.356	3.368
**SE**	0.346	0.345	0.344	0.343	0.342	0.341	0.340	0.339
**Observations**	139	139	139	139	139	139	139	139

*** p < 0.01, ** p < 0.05, * p < 0.10

Table K. Interrupted time-series with two intervention points for high-income countries (HICs). Results presented for 0 to 15-day lags.

	0-day lag	1-day lag	2-day lag	3-day lag	4-day lag	5-day lag	6-day lag	7-day lag
**Time**	-0.236***	-0.254***	-0.252***	-0.234***	-0.214***	-0.191***	-0.170***	-0.152***
**LCI**	-0.345	-0.338	-0.319	-0.299	-0.278	-0.255	-0.232	-0.210
**UCI**	-0.127	-0.170	-0.184	-0.169	-0.150	-0.128	-0.108	-0.0933
**SE**	0.0550	0.0423	0.0341	0.0330	0.0323	0.0322	0.0313	0.0295
**Feb 5 level change**	0.583	1.066**	1.423***	1.642***	1.811***	1.922***	2.002***	2.076***
**LCI**	-0.287	0.214	0.577	0.739	0.846	0.892	0.919	0.958
**UCI**	1.453	1.919	2.269	2.546	2.775	2.952	3.085	3.194
**SE**	0.440	0.431	0.427	0.457	0.487	0.521	0.547	0.565
**Feb 5 slope change**	0.256***	0.270***	0.264***	0.242***	0.216***	0.189***	0.163***	0.140***
**LCI**	0.146	0.185	0.194	0.173	0.148	0.121	0.0957	0.0751
**UCI**	0.366	0.356	0.334	0.310	0.284	0.258	0.231	0.205
**SE**	0.0557	0.0433	0.0355	0.0347	0.0344	0.0346	0.0341	0.0329
**Mar 19 level change**	-1.175***	-1.164***	-1.153***	-1.105***	-1.042***	-0.964***	-0.894***	-0.831***
**LCI**	-1.599	-1.548	-1.500	-1.436	-1.369	-1.303	-1.248	-1.203
**UCI**	-0.751	-0.781	-0.807	-0.774	-0.714	-0.624	-0.539	-0.458
**SE**	0.214	0.194	0.175	0.167	0.166	0.172	0.179	0.188
**Mar 19 slope change**	-0.0276***	-0.0230**	-0.0181*	-0.0130	-0.00762	-0.00221	0.00302	0.00821
**LCI**	-0.0452	-0.0416	-0.0379	-0.0344	-0.0308	-0.0274	-0.0240	-0.0205
**UCI**	-0.00999	-0.00435	0.00162	0.00844	0.0156	0.0230	0.0300	0.0370
**SE**	0.00890	0.00941	0.00999	0.0108	0.0117	0.0127	0.0137	0.0145
**Constant**	3.204***	3.251***	3.245***	3.186***	3.111***	3.022***	2.930***	2.844***
**LCI**	2.642	2.744	2.764	2.704	2.626	2.525	2.421	2.326
**UCI**	3.766	3.758	3.726	3.667	3.596	3.519	3.439	3.363
**SE**	0.284	0.256	0.243	0.243	0.245	0.251	0.257	0.262
**Observations**	135	135	135	135	135	135	135	135

	8-day lag	9-day lag	10-day lag	11-day lag	12-day lag	13-day lag	14-day lag	15-day lag
**Time**	-0.139***	-0.132***	-0.126***	-0.117***	-0.103***	-0.0851***	-0.0713***	-0.0641***
**LCI**	-0.192	-0.178	-0.167	-0.157	-0.146	-0.134	-0.120	-0.108
**UCI**	-0.0871	-0.0853	-0.0841	-0.0777	-0.0598	-0.0367	-0.0231	-0.0202
**SE**	0.0265	0.0234	0.0210	0.0199	0.0217	0.0245	0.0244	0.0222
**Feb 5 level change**	2.207***	2.396***	2.617***	2.789***	2.818***	2.729***	2.672***	2.738***
**LCI**	1.084	1.285	1.529	1.718	1.713	1.555	1.482	1.601
**UCI**	3.331	3.508	3.706	3.860	3.923	3.903	3.862	3.876
**SE**	0.568	0.562	0.550	0.542	0.558	0.593	0.601	0.575
**Feb 5 slope change**	0.123***	0.109***	0.0960***	0.0805***	0.0604**	0.0385	0.0204	0.00782
**LCI**	0.0624	0.0535	0.0442	0.0304	0.00718	-0.0197	-0.0378	-0.0464
**UCI**	0.183	0.165	0.148	0.131	0.114	0.0966	0.0787	0.0621
**SE**	0.0305	0.0281	0.0262	0.0253	0.0269	0.0294	0.0294	0.0274
**Mar 19 level change**	-0.764***	-0.680***	-0.556***	-0.416**	-0.290	-0.192	-0.101	0.0106
**LCI**	-1.151	-1.077	-0.960	-0.827	-0.718	-0.643	-0.570	-0.457
**UCI**	-0.378	-0.284	-0.152	-0.00472	0.138	0.259	0.368	0.478
**SE**	0.195	0.200	0.204	0.208	0.216	0.228	0.237	0.236
**Mar 19 slope change**	0.0140	0.0206	0.0280*	0.0354**	0.0414***	0.0459***	0.0505***	0.0563***
**LCI**	-0.0161	-0.0103	-0.00298	0.00449	0.0101	0.0138	0.0179	0.0244
**UCI**	0.0441	0.0514	0.0590	0.0663	0.0727	0.0781	0.0831	0.0881
**SE**	0.0152	0.0156	0.0157	0.0156	0.0158	0.0162	0.0165	0.0161
**Constant**	2.783***	2.741***	2.707***	2.656***	2.565***	2.448***	2.351***	2.298***
**LCI**	2.267	2.234	2.208	2.153	2.029	1.862	1.744	1.698
**UCI**	3.298	3.248	3.206	3.160	3.102	3.033	2.958	2.898
**SE**	0.261	0.256	0.252	0.254	0.271	0.296	0.307	0.303
**Observations**	135	135	135	135	135	135	135	135

*** $p < 0.01$, ** $p < 0.05$, * $p < 0.10$

Table L. Interrupted time-series with one intervention point for high-income countries (HICs). Results presented for 0 to 15-day lags.

	0-day lag	1-day lag	2-day lag	3-day lag	4-day lag	5-day lag	6-day lag	7-day lag
**Time**	0.00816	0.00782	0.00752	0.00666	0.00569	0.00456	0.00358	0.00276
**LCI**	-0.00475	-0.00455	-0.00438	-0.00485	-0.00552	-0.00641	-0.00714	-0.00772
**UCI**	0.0211	0.0202	0.0194	0.0182	0.0169	0.0155	0.0143	0.0132
**SE**	0.00652	0.00625	0.00601	0.00582	0.00566	0.00554	0.00542	0.00530
**Feb 5 level change**	-0.963***	-1.005***	-1.051***	-1.061***	-1.061***	-1.047***	-1.040***	-1.042***
**LCI**	-1.415	-1.431	-1.451	-1.442	-1.426	-1.400	-1.381	-1.368
**UCI**	-0.510	-0.579	-0.651	-0.680	-0.696	-0.694	-0.700	-0.717
**SE**	0.229	0.216	0.202	0.192	0.184	0.179	0.172	0.164
**Feb 5 slope change**	-0.0156**	-0.0146**	-0.0134**	-0.0120**	-0.0105*	-0.00897	-0.00750	-0.00610
**LCI**	-0.0289	-0.0272	-0.0256	-0.0238	-0.0219	-0.0202	-0.0185	-0.0168
**UCI**	-0.00242	-0.00189	-0.00129	-0.000251	0.000928	0.00224	0.00346	0.00460
**SE**	0.00669	0.00640	0.00614	0.00594	0.00578	0.00567	0.00554	0.00541
**Constant**	1.914***	1.920***	1.925***	1.941***	1.959***	1.981***	2.000***	2.016***
**LCI**	1.373	1.379	1.385	1.401	1.419	1.440	1.459	1.475
**UCI**	2.455	2.461	2.466	2.481	2.500	2.521	2.540	2.556
**SE**	0.274	0.273	0.273	0.273	0.273	0.273	0.273	0.273
**Observations**	135	135	135	135	135	135	135	135
	**8-day lag**	**9-day lag**	**10-day lag**	**11-day lag**	**12-day lag**	**13-day lag**	**14-day lag**	**15-day lag**
**Time**	0.00207	0.00134	0.000342	-0.000790	-0.00191	-0.00288	-0.00373	-0.00451
**LCI**	-0.00818	-0.00870	-0.00958	-0.0106	-0.0117	-0.0126	-0.0133	-0.0139
**UCI**	0.0123	0.0114	0.0103	0.00907	0.00788	0.00680	0.00583	0.00491
**SE**	0.00518	0.00508	0.00502	0.00498	0.00495	0.00490	0.00483	0.00476
**Feb 5 level change**	-1.051***	-1.057***	-1.037***	-1.002***	-0.963***	-0.931***	-0.906***	-0.883***
**LCI**	-1.360	-1.349	-1.322	-1.284	-1.243	-1.207	-1.175	-1.144
**UCI**	-0.743	-0.764	-0.752	-0.720	-0.683	-0.655	-0.638	-0.621
**SE**	0.156	0.148	0.144	0.143	0.142	0.139	0.136	0.132
**Feb 5 slope change**	-0.00473	-0.00334	-0.00191	-0.000491	0.000855	0.00212	0.00333	0.00450
**LCI**	-0.0152	-0.0135	-0.0119	-0.0105	-0.00904	-0.00767	-0.00632	-0.00500
**UCI**	0.00569	0.00682	0.00812	0.00947	0.0108	0.0119	0.0130	0.0140
**SE**	0.00527	0.00514	0.00507	0.00504	0.00500	0.00495	0.00488	0.00481
**Constant**	2.030***	2.044***	2.065***	2.089***	2.113***	2.134***	2.152***	2.170***
**LCI**	1.489	1.504	1.524	1.547	1.571	1.592	1.610	1.628
**UCI**	2.570	2.585	2.606	2.630	2.655	2.676	2.695	2.712
**SE**	0.273	0.273	0.273	0.274	0.274	0.274	0.274	0.274
**Observations**	135	135	135	135	135	135	135	135

*** p < 0.01, ** p < 0.05, * p < 0.10

Table M. Interrupted time-series with two intervention points for low- and middle-income countries (LMICs), excluding China. Results presented for 0 to 15-day lags.

	0-day lag	1-day lag	2-day lag	3-day lag	4-day lag	5-day lag	6-day lag	7-day lag
**Time**	-0.113***	-0.119***	-0.0940***	-0.0777***	-0.0636**	-0.0702***	-0.0738***	-0.0754***
**LCI**	-0.165	-0.163	-0.151	-0.131	-0.112	-0.113	-0.110	-0.106
**UCI**	-0.0602	-0.0742	-0.0372	-0.0246	-0.0146	-0.0273	-0.0374	-0.0446
**SE**	0.0266	0.0225	0.0287	0.0268	0.0247	0.0216	0.0184	0.0156
**Feb 5 level change**	1.199***	1.517***	1.467***	1.469***	1.456***	1.747***	2.022***	2.290***
**LCI**	0.399	0.690	0.514	0.469	0.417	0.694	0.970	1.249
**UCI**	1.999	2.345	2.420	2.468	2.495	2.800	3.073	3.331
**SE**	0.404	0.418	0.482	0.505	0.525	0.532	0.531	0.526
**Feb 5 slope change**	0.117***	0.118***	0.0893***	0.0695**	0.0526*	0.0538**	0.0515**	0.0463**
**LCI**	0.0602	0.0676	0.0277	0.0108	-0.00321	0.00280	0.00531	0.00398
**UCI**	0.174	0.167	0.151	0.128	0.108	0.105	0.0977	0.0885
**SE**	0.0289	0.0252	0.0311	0.0297	0.0282	0.0258	0.0233	0.0214
**Mar 19 level change**	-0.850***	-0.753***	-0.687***	-0.633**	-0.607**	-0.540**	-0.450**	-0.321
**LCI**	-1.422	-1.276	-1.187	-1.114	-1.079	-0.995	-0.893	-0.758
**UCI**	-0.278	-0.229	-0.188	-0.151	-0.134	-0.0849	-0.00668	0.116
**SE**	0.289	0.265	0.253	0.243	0.239	0.230	0.224	0.221
**Mar 19 slope change**	-0.0103	-0.00429	-0.000629	0.00314	0.00648	0.0125	0.0190	0.0261*
**LCI**	-0.0326	-0.0268	-0.0245	-0.0222	-0.0205	-0.0153	-0.00947	-0.00277
**UCI**	0.0120	0.0182	0.0232	0.0285	0.0335	0.0403	0.0475	0.0550
**SE**	0.0113	0.0114	0.0121	0.0128	0.0137	0.0141	0.0144	0.0146
**Constant**	2.240***	2.262***	2.163***	2.092***	2.027***	2.060***	2.079***	2.088***
**LCI**	1.864	1.919	1.769	1.692	1.621	1.688	1.738	1.770
**UCI**	2.616	2.606	2.557	2.493	2.432	2.431	2.420	2.406
**SE**	0.190	0.174	0.199	0.202	0.205	0.188	0.172	0.161
**Observations**	135	135	135	135	135	135	135	135

	8-day lag	9-day lag	10-day lag	11-day lag	12-day lag	13-day lag	14-day lag	15-day lag
**Time**	-0.0755***	-0.0698***	-0.0642***	-0.0584***	-0.0563***	-0.0525***	-0.0457***	-0.0226
**LCI**	-0.102	-0.0957	-0.0893	-0.0830	-0.0784	-0.0734	-0.0680	-0.0684
**UCI**	-0.0489	-0.0438	-0.0391	-0.0339	-0.0343	-0.0315	-0.0233	0.0231
**SE**	0.0134	0.0131	0.0127	0.0124	0.0112	0.0106	0.0113	0.0231
**Feb 5 level change**	2.543***	2.680***	2.808***	2.921***	3.116***	3.266***	3.325***	2.878***
**LCI**	1.524	1.666	1.814	1.960	2.251	2.520	2.692	1.892
**UCI**	3.561	3.693	3.801	3.883	3.982	4.012	3.959	3.863
**SE**	0.515	0.512	0.502	0.486	0.437	0.377	0.320	0.498
**Feb 5 slope change**	0.0393**	0.0275	0.0160	0.00416	-0.00517	-0.0160	-0.0287**	-0.0512**
**LCI**	0.000133	-0.0114	-0.0220	-0.0329	-0.0385	-0.0456	-0.0562	-0.1000
**UCI**	0.0785	0.0663	0.0540	0.0412	0.0282	0.0137	-0.00111	-0.00246
**SE**	0.0198	0.0196	0.0192	0.0187	0.0169	0.0150	0.0139	0.0246
**Mar 19 level change**	-0.188	-0.0687	0.0445	0.170	0.324	0.482***	0.626***	0.665***
**LCI**	-0.618	-0.501	-0.389	-0.260	-0.0793	0.121	0.315	0.351
**UCI**	0.242	0.364	0.478	0.601	0.728	0.844	0.936	0.979
**SE**	0.217	0.219	0.219	0.218	0.204	0.183	0.157	0.159
**Mar 19 slope change**	0.0334**	0.0397***	0.0460***	0.0523***	0.0596***	0.0665***	0.0724***	0.0719***
**LCI**	0.00458	0.0108	0.0174	0.0245	0.0345	0.0454	0.0562	0.0550
**UCI**	0.0622	0.0687	0.0746	0.0800	0.0846	0.0876	0.0885	0.0887
**SE**	0.0146	0.0146	0.0145	0.0140	0.0127	0.0106	0.00816	0.00854
**Constant**	2.089***	2.052***	2.015***	1.975***	1.959***	1.930***	1.876***	1.684***
**LCI**	1.787	1.745	1.705	1.659	1.652	1.623	1.552	1.188
**UCI**	2.391	2.359	2.326	2.291	2.267	2.237	2.199	2.179
**SE**	0.153	0.155	0.157	0.160	0.155	0.155	0.164	0.251
**Observations**	135	135	135	135	135	135	135	135

*** p < 0.01, ** p < 0.05, * p < 0.10

Table N. Interrupted time-series with one intervention point for low- and middle-income countries (LMICs), excluding China. Results presented for 0 to 15-day lags.

	0-day lag	1-day lag	2-day lag	3-day lag	4-day lag	5-day lag	6-day lag	7-day lag
**Time**	0.0109*	0.00966	0.00844	0.00749	0.00688	0.00632	0.00559	0.00457
**LCI**	-0.00155	-0.00199	-0.00252	-0.00283	-0.00284	-0.00288	-0.00318	-0.00390
**UCI**	0.0233	0.0213	0.0194	0.0178	0.0166	0.0155	0.0144	0.0130
**SE**	0.00629	0.00589	0.00554	0.00522	0.00492	0.00465	0.00443	0.00428
**Feb 5 level change**	-0.948***	-0.926***	-0.902***	-0.892***	-0.907***	-0.924***	-0.926***	-0.901***
**LCI**	-1.524	-1.474	-1.424	-1.389	-1.376	-1.366	-1.345	-1.302
**UCI**	-0.372	-0.379	-0.380	-0.396	-0.438	-0.481	-0.507	-0.499
**SE**	0.291	0.277	0.264	0.251	0.237	0.224	0.212	0.203
**Feb 5 slope change**	-0.0167***	-0.0152**	-0.0138**	-0.0125**	-0.0113**	-0.0101**	-0.00889**	-0.00762*
**LCI**	-0.0292	-0.0270	-0.0249	-0.0230	-0.0212	-0.0195	-0.0178	-0.0162
**UCI**	-0.00407	-0.00338	-0.00263	-0.00197	-0.00141	-0.000792	-2.59e-05	0.000941
**SE**	0.00636	0.00597	0.00563	0.00532	0.00500	0.00472	0.00448	0.00433
**Constant**	1.759***	1.783***	1.806***	1.825***	1.837***	1.849***	1.864***	1.885***
**LCI**	1.351	1.374	1.395	1.413	1.423	1.433	1.446	1.463
**UCI**	2.168	2.192	2.217	2.238	2.251	2.264	2.282	2.308
**SE**	0.207	0.207	0.208	0.209	0.209	0.210	0.211	0.213
**Observations**	135	135	135	135	135	135	135	135
	**8-day lag**	**9-day lag**	**10-day lag**	**11-day lag**	**12-day lag**	**13-day lag**	**14-day lag**	**15-day lag**
**Time**	0.00360	0.00270	0.00192	0.00106	0.000159	-0.000761	-0.00163	-0.00240
**LCI**	-0.00460	-0.00527	-0.00584	-0.00653	-0.00732	-0.00816	-0.00895	-0.00963
**UCI**	0.0118	0.0107	0.00967	0.00865	0.00764	0.00663	0.00569	0.00483
**SE**	0.00415	0.00403	0.00392	0.00384	0.00378	0.00374	0.00370	0.00366
**Feb 5 level change**	-0.876***	-0.853***	-0.836***	-0.810***	-0.774***	-0.731***	-0.688***	-0.650***
**LCI**	-1.260	-1.221	-1.187	-1.147	-1.099	-1.046	-0.994	-0.945
**UCI**	-0.492	-0.485	-0.485	-0.473	-0.449	-0.416	-0.383	-0.354
**SE**	0.194	0.186	0.177	0.170	0.164	0.159	0.154	0.149
**Feb 5 slope change**	-0.00642	-0.00526	-0.00415	-0.00309	-0.00210	-0.00120	-0.000372	0.000388
**LCI**	-0.0147	-0.0133	-0.0120	-0.0108	-0.00966	-0.00869	-0.00779	-0.00696
**UCI**	0.00189	0.00280	0.00368	0.00458	0.00546	0.00630	0.00705	0.00773
**SE**	0.00420	0.00408	0.00396	0.00388	0.00382	0.00379	0.00375	0.00371
**Constant**	1.906***	1.925***	1.943***	1.962***	1.982***	2.003***	2.024***	2.042***
**LCI**	1.480	1.495	1.509	1.524	1.541	1.558	1.574	1.589
**UCI**	2.332	2.356	2.376	2.399	2.424	2.449	2.473	2.495
**SE**	0.216	0.217	0.219	0.221	0.223	0.225	0.227	0.229
**Observations**	135	135	135	135	135	135	135	135

*** p < 0.01, ** p < 0.05, * p < 0.10

Table O. Interrupted time-series with two intervention points for low- and middle-income countries (LMICs), excluding China. Results presented for 0 to 15-day lags.

	0-day lag	1-day lag	2-day lag	3-day lag	4-day lag	5-day lag	6-day lag	7-day lag
**Time**	-0.226***	-0.226***	-0.225***	-0.220***	-0.214***	-0.207***	-0.203***	-0.173***
**LCI**	-0.322	-0.310	-0.300	-0.288	-0.277	-0.265	-0.256	-0.243
**UCI**	-0.131	-0.142	-0.149	-0.151	-0.151	-0.149	-0.149	-0.104
**SE**	0.0481	0.0426	0.0382	0.0347	0.0318	0.0294	0.0269	0.0351
**Feb 5 level change**	-0.460	-0.272	-0.0722	0.0855	0.231	0.361	0.549*	0.279
**LCI**	-1.033	-0.823	-0.616	-0.465	-0.334	-0.224	-0.0423	-0.578
**UCI**	0.113	0.278	0.471	0.636	0.795	0.945	1.140	1.135
**SE**	0.290	0.278	0.275	0.278	0.285	0.295	0.299	0.433
**Feb 5 slope change**	0.242***	0.243***	0.243***	0.240***	0.235***	0.229***	0.225***	0.199***
**LCI**	0.146	0.158	0.167	0.170	0.170	0.169	0.169	0.128
**UCI**	0.338	0.329	0.320	0.309	0.299	0.289	0.280	0.270
**SE**	0.0485	0.0431	0.0387	0.0354	0.0326	0.0304	0.0281	0.0359
**Mar 19 level change**	-0.00840	-0.0818	-0.137	-0.204*	-0.289**	-0.370***	-0.418***	-0.494***
**LCI**	-0.206	-0.283	-0.346	-0.423	-0.520	-0.612	-0.669	-0.742
**UCI**	0.189	0.119	0.0718	0.0155	-0.0582	-0.129	-0.166	-0.247
**SE**	0.0998	0.102	0.106	0.111	0.117	0.122	0.127	0.125
**Mar 19 slope change**	-0.0212***	-0.0229***	-0.0239***	-0.0248***	-0.0256***	-0.0260***	-0.0252***	-0.0282***
**LCI**	-0.0328	-0.0351	-0.0368	-0.0385	-0.0401	-0.0414	-0.0413	-0.0435
**UCI**	-0.00957	-0.0107	-0.0110	-0.0112	-0.0111	-0.0106	-0.00918	-0.0128
**SE**	0.00587	0.00616	0.00652	0.00691	0.00734	0.00776	0.00812	0.00777
**Constant**	3.909***	3.908***	3.903***	3.881***	3.853***	3.818***	3.797***	3.631***
**LCI**	3.072	3.100	3.124	3.128	3.125	3.111	3.113	2.900
**UCI**	4.747	4.715	4.682	4.633	4.581	4.525	4.481	4.361
**SE**	0.423	0.408	0.394	0.380	0.368	0.357	0.346	0.369
**Observations**	135	135	135	135	135	135	135	135
	**8-day lag**	**9-day lag**	**10-day lag**	**11-day lag**	**12-day lag**	**13-day lag**	**14-day lag**	**15-day lag**
**Time**	-0.142***	-0.120***	-0.120***	-0.123***	-0.124***	-0.124***	-0.124***	-0.124***
**LCI**	-0.221	-0.195	-0.184	-0.179	-0.173	-0.168	-0.164	-0.159
**UCI**	-0.0633	-0.0457	-0.0555	-0.0660	-0.0737	-0.0797	-0.0843	-0.0878
**SE**	0.0397	0.0377	0.0325	0.0286	0.0252	0.0224	0.0200	0.0181
**Feb 5 level change**	-0.118	-0.404	-0.277	-0.0565	0.146	0.352	0.561*	0.776***
**LCI**	-1.123	-1.363	-1.132	-0.843	-0.574	-0.310	-0.0514	0.208
**UCI**	0.887	0.555	0.579	0.730	0.865	1.015	1.174	1.343
**SE**	0.508	0.485	0.432	0.397	0.364	0.335	0.310	0.287
**Feb 5 slope change**	0.172***	0.154***	0.154***	0.155***	0.154***	0.152***	0.149***	0.145***
**LCI**	0.0923	0.0793	0.0892	0.0980	0.104	0.107	0.108	0.109
**UCI**	0.251	0.229	0.218	0.212	0.204	0.197	0.190	0.182
**SE**	0.0402	0.0378	0.0326	0.0288	0.0255	0.0227	0.0205	0.0186
**Mar 19 level change**	-0.596***	-0.687***	-0.718***	-0.714***	-0.693***	-0.654***	-0.606***	-0.555***
**LCI**	-0.800	-0.839	-0.876	-0.886	-0.884	-0.863	-0.826	-0.775
**UCI**	-0.392	-0.535	-0.561	-0.541	-0.502	-0.445	-0.387	-0.334
**SE**	0.103	0.0769	0.0796	0.0872	0.0966	0.106	0.111	0.111
**Mar 19 slope change**	-0.0326***	-0.0364***	-0.0362***	-0.0344***	-0.0324***	-0.0299***	-0.0270***	-0.0238***
**LCI**	-0.0444	-0.0427	-0.0422	-0.0411	-0.0399	-0.0381	-0.0358	-0.0327
**UCI**	-0.0209	-0.0302	-0.0301	-0.0277	-0.0249	-0.0216	-0.0183	-0.0150
**SE**	0.00593	0.00314	0.00307	0.00339	0.00378	0.00417	0.00443	0.00447
**Constant**	3.442***	3.304***	3.301***	3.321***	3.328***	3.331***	3.331***	3.329***
**LCI**	2.664	2.526	2.568	2.628	2.668	2.698	2.719	2.735
**UCI**	4.220	4.083	4.034	4.014	3.989	3.965	3.943	3.922
**SE**	0.393	0.394	0.370	0.350	0.334	0.320	0.309	0.300
**Observations**	135	135	135	135	135	135	135	135

*** $p < 0.01$, ** $p < 0.05$, * $p < 0.10$

Table P. Interrupted time-series with one intervention point for low- and middle-income countries (LMICs), including China. Results presented for 0 to 15-day lags.

	**0-day lag**	**1-day lag**	**2-day lag**	**3-day lag**	**4-day lag**	**5-day lag**	**6-day lag**	**7-day lag**
**Time**	-0.0211***	-0.0196**	-0.0184**	-0.0170**	-0.0155**	-0.0141*	-0.0130*	-0.0124*
**LCI**	-0.0369	-0.0350	-0.0334	-0.0317	-0.0300	-0.0284	-0.0269	-0.0259
**UCI**	-0.00536	-0.00422	-0.00333	-0.00231	-0.00103	0.000117	0.000876	0.00113
**SE**	0.00797	0.00779	0.00760	0.00744	0.00732	0.00720	0.00703	0.00683
**Feb 5 level change**	0.652***	0.597***	0.550***	0.491**	0.413**	0.335*	0.275	0.246
**LCI**	0.251	0.201	0.161	0.108	0.0330	-0.0387	-0.0900	-0.109
**UCI**	1.053	0.992	0.938	0.875	0.793	0.710	0.641	0.601
**SE**	0.203	0.200	0.196	0.194	0.192	0.189	0.185	0.179
**Feb 5 slope change**	0.0155*	0.0142*	0.0131*	0.0121	0.0112	0.0104	0.00981	0.00939
**LCI**	-0.000412	-0.00132	-0.00204	-0.00274	-0.00343	-0.00393	-0.00416	-0.00418
**UCI**	0.0313	0.0297	0.0283	0.0270	0.0258	0.0247	0.0238	0.0230
**SE**	0.00802	0.00785	0.00767	0.00751	0.00738	0.00724	0.00706	0.00686
**Constant**	2.011***	1.983***	1.958***	1.932***	1.902***	1.873***	1.851***	1.837***
**LCI**	1.407	1.380	1.358	1.333	1.303	1.275	1.255	1.246
**UCI**	2.615	2.585	2.559	2.531	2.500	2.471	2.447	2.428
**SE**	0.305	0.305	0.303	0.303	0.303	0.302	0.301	0.299
**Observations**	135	135	135	135	135	135	135	135

	**8-day lag**	**9-day lag**	**10-day lag**	**11-day lag**	**12-day lag**	**13-day lag**	**14-day lag**	**15-day lag**
**Time**	-0.0118*	-0.0113*	-0.0107*	-0.0103*	-0.0101*	-0.00995*	-0.00983*	-0.00968*
**LCI**	-0.0249	-0.0240	-0.0232	-0.0225	-0.0219	-0.0214	-0.0210	-0.0206
**UCI**	0.00134	0.00151	0.00173	0.00177	0.00169	0.00152	0.00134	0.00120
**SE**	0.00664	0.00646	0.00629	0.00612	0.00596	0.00580	0.00565	0.00550
**Feb 5 level change**	0.218	0.192	0.162	0.144	0.137	0.139	0.143	0.145
**LCI**	-0.127	-0.144	-0.165	-0.175	-0.174	-0.165	-0.154	-0.146
**UCI**	0.564	0.528	0.489	0.463	0.448	0.443	0.441	0.436
**SE**	0.175	0.170	0.165	0.161	0.157	0.154	0.150	0.147
**Feb 5 slope change**	0.00904	0.00876	0.00853	0.00835	0.00819	0.00802	0.00785	0.00769
**LCI**	-0.00415	-0.00408	-0.00397	-0.00382	-0.00365	-0.00351	-0.00338	-0.00327
**UCI**	0.0222	0.0216	0.0210	0.0205	0.0200	0.0196	0.0191	0.0186
**SE**	0.00667	0.00649	0.00632	0.00615	0.00599	0.00583	0.00568	0.00554
**Constant**	1.825***	1.813***	1.801***	1.793***	1.787***	1.784***	1.781***	1.778***
**LCI**	1.238	1.232	1.224	1.221	1.221	1.223	1.226	1.228
**UCI**	2.411	2.395	2.378	2.365	2.354	2.345	2.336	2.327
**SE**	0.296	0.294	0.292	0.289	0.286	0.284	0.281	0.278
**Observations**	135	135	135	135	135	135	135	135

*** $p < 0.01$, ** $p < 0.05$, * $p < 0.10$

Table Q. Global interrupted time-series results with one intervention point, excluding China. Results presented for 0 to 15-day lags.

	0-day lag	1-day lag	2-day lag	3-day lag	4-day lag	5-day lag	6-day lag	7-day lag
**Time**	0.00908	0.00815	0.00713	0.00598	0.00497	0.00412	0.00342	0.00271
**LCI**	-0.00258	-0.00310	-0.00380	-0.00469	-0.00546	-0.00606	-0.00652	-0.00704
**UCI**	0.0207	0.0194	0.0181	0.0167	0.0154	0.0143	0.0134	0.0125
**SE**	0.00589	0.00569	0.00552	0.00539	0.00527	0.00515	0.00503	0.00493
**Feb 5 level change**	-1.049***	-1.054***	-1.051***	-1.036***	-1.026***	-1.026***	-1.035***	-1.040***
**LCI**	-1.463	-1.448	-1.427	-1.398	-1.374	-1.358	-1.348	-1.336
**UCI**	-0.634	-0.660	-0.675	-0.673	-0.679	-0.695	-0.721	-0.744
**SE**	0.209	0.199	0.190	0.183	0.176	0.168	0.158	0.150
**Feb 5 slope change**	-0.0141**	-0.0126**	-0.0111*	-0.00954*	-0.00808	-0.00668	-0.00533	-0.00397
**LCI**	-0.0259	-0.0241	-0.0222	-0.0204	-0.0187	-0.0171	-0.0154	-0.0138
**UCI**	-0.00217	-0.00111	6.15e-05	0.00136	0.00258	0.00371	0.00478	0.00589
**SE**	0.00601	0.00580	0.00563	0.00551	0.00539	0.00525	0.00511	0.00498
**Constant**	1.856***	1.874***	1.893***	1.914***	1.934***	1.951***	1.965***	1.979***
**LCI**	1.328	1.345	1.363	1.384	1.402	1.418	1.432	1.446
**UCI**	2.385	2.402	2.422	2.445	2.466	2.483	2.497	2.513
**SE**	0.267	0.267	0.268	0.268	0.269	0.269	0.269	0.270
**Observations**	135	135	135	135	135	135	135	135
	**8-day lag**	**9-day lag**	**10-day lag**	**11-day lag**	**12-day lag**	**13-day lag**	**14-day lag**	**15-day lag**
**Time**	0.00173	0.000597	-0.000510	-0.00149	-0.00233	-0.00313	-0.00392	-0.00476
**LCI**	-0.00790	-0.00897	-0.0100	-0.0109	-0.0116	-0.0123	-0.0130	-0.0138
**UCI**	0.0114	0.0102	0.00900	0.00794	0.00698	0.00607	0.00517	0.00425
**SE**	0.00487	0.00484	0.00481	0.00476	0.00471	0.00465	0.00460	0.00455
**Feb 5 level change**	-1.022***	-0.987***	-0.949***	-0.916***	-0.892***	-0.867***	-0.838***	-0.801***
**LCI**	-1.308	-1.269	-1.227	-1.189	-1.157	-1.124	-1.090	-1.048
**UCI**	-0.736	-0.705	-0.670	-0.644	-0.627	-0.609	-0.587	-0.554
**SE**	0.145	0.143	0.141	0.138	0.134	0.130	0.127	0.125
**Feb 5 slope change**	-0.00255	-0.00115	0.000172	0.00142	0.00261	0.00376	0.00486	0.00588
**LCI**	-0.0123	-0.0108	-0.00944	-0.00810	-0.00678	-0.00551	-0.00430	-0.00320
**UCI**	0.00717	0.00851	0.00978	0.0109	0.0120	0.0130	0.0140	0.0150
**SE**	0.00491	0.00489	0.00486	0.00481	0.00475	0.00469	0.00463	0.00459
**Constant**	1.999***	2.023***	2.047***	2.068***	2.086***	2.104***	2.122***	2.142***
**LCI**	1.465	1.487	1.509	1.529	1.548	1.565	1.583	1.602
**UCI**	2.534	2.559	2.584	2.606	2.625	2.644	2.662	2.681
**SE**	0.270	0.271	0.272	0.272	0.272	0.273	0.273	0.273
**Observations**	135	135	135	135	135	135	135	135

*** p < 0.01, ** p < 0.05, * p < 0.10

Table R. Global interrupted time-series results with two intervention points, including China. Results presented for 0 to 15-day lags.

	0-day lag	1-day lag	2-day lag	3-day lag	4-day lag	5-day lag	6-day lag	7-day lag
**Time**	-0.226***	-0.225***	-0.219***	-0.213***	-0.206***	-0.202***	-0.173***	-0.142***
**LCI**	-0.309	-0.299	-0.287	-0.276	-0.264	-0.255	-0.242	-0.220
**UCI**	-0.143	-0.150	-0.152	-0.151	-0.149	-0.150	-0.104	-0.0635
**SE**	0.0420	0.0377	0.0342	0.0314	0.0291	0.0266	0.0348	0.0395
**Feb 5 level change**	-0.291	-0.0730	0.113	0.300	0.471	0.694**	0.439	0.0574
**LCI**	-0.851	-0.627	-0.450	-0.277	-0.126	0.0914	-0.426	-0.956
**UCI**	0.268	0.482	0.675	0.877	1.068	1.297	1.305	1.070
**SE**	0.283	0.280	0.284	0.292	0.302	0.305	0.437	0.512
**Feb 5 slope change**	0.257***	0.256***	0.251***	0.244***	0.236***	0.230***	0.204***	0.177***
**LCI**	0.173	0.180	0.182	0.180	0.176	0.174	0.133	0.0975
**UCI**	0.341	0.332	0.320	0.308	0.296	0.286	0.275	0.256
**SE**	0.0426	0.0383	0.0351	0.0324	0.0303	0.0281	0.0359	0.0402
**Mar 19 level change**	-0.684***	-0.750***	-0.799***	-0.823***	-0.844***	-0.859***	-0.951***	-1.065***
**LCI**	-0.913	-0.986	-1.047	-1.092	-1.131	-1.155	-1.246	-1.330
**UCI**	-0.455	-0.515	-0.551	-0.553	-0.557	-0.563	-0.657	-0.801
**SE**	0.116	0.119	0.126	0.136	0.145	0.150	0.149	0.134
**Mar 19 slope change**	-0.0358***	-0.0359***	-0.0355***	-0.0343***	-0.0327***	-0.0303***	-0.0326***	-0.0365***
**LCI**	-0.0494	-0.0503	-0.0508	-0.0506	-0.0500	-0.0482	-0.0500	-0.0505
**UCI**	-0.0222	-0.0215	-0.0202	-0.0180	-0.0155	-0.0123	-0.0151	-0.0225
**SE**	0.00687	0.00727	0.00773	0.00825	0.00872	0.00906	0.00881	0.00707
**Constant**	3.903***	3.898***	3.875***	3.847***	3.812***	3.791***	3.625***	3.437***
**LCI**	3.106	3.129	3.132	3.128	3.114	3.115	2.902	2.665
**UCI**	4.701	4.667	4.619	4.567	4.510	4.467	4.348	4.208
**SE**	0.403	0.389	0.376	0.364	0.353	0.342	0.366	0.390
**Observations**	135	135	135	135	135	135	135	135
	**8-day lag**	**9-day lag**	**10-day lag**	**11-day lag**	**12-day lag**	**13-day lag**	**14-day lag**	**15-day lag**
**Time**	-0.120***	-0.120***	-0.122***	-0.123***	-0.124***	-0.124***	-0.123***	-0.120***
**LCI**	-0.194	-0.183	-0.179	-0.173	-0.168	-0.163	-0.159	-0.153
**UCI**	-0.0458	-0.0556	-0.0660	-0.0737	-0.0797	-0.0842	-0.0875	-0.0873
**SE**	0.0375	0.0323	0.0284	0.0250	0.0222	0.0199	0.0180	0.0167
**Feb 5 level change**	-0.204	-0.0361	0.225	0.464	0.703*	0.944***	1.191***	1.379***
**LCI**	-1.175	-0.912	-0.589	-0.292	-0.00142	0.286	0.579	0.797
**UCI**	0.767	0.839	1.039	1.219	1.407	1.602	1.804	1.962
**SE**	0.491	0.442	0.411	0.382	0.356	0.332	0.309	0.294
**Feb 5 slope change**	0.158***	0.156***	0.155***	0.152***	0.148***	0.144***	0.138***	0.130***
**LCI**	0.0835	0.0909	0.0973	0.101	0.102	0.102	0.100	0.0950
**UCI**	0.233	0.221	0.212	0.203	0.194	0.185	0.176	0.166
**SE**	0.0378	0.0328	0.0291	0.0258	0.0232	0.0210	0.0191	0.0178
**Mar 19 level change**	-1.137***	-1.115***	-1.063***	-1.012***	-0.961***	-0.899***	-0.823***	-0.740***
**LCI**	-1.385	-1.390	-1.358	-1.316	-1.263	-1.194	-1.104	-1.013
**UCI**	-0.890	-0.840	-0.768	-0.708	-0.659	-0.605	-0.543	-0.467
**SE**	0.125	0.139	0.149	0.154	0.153	0.149	0.142	0.138
**Mar 19 slope change**	-0.0392***	-0.0368***	-0.0329***	-0.0288***	-0.0244***	-0.0195***	-0.0140**	-0.00897
**LCI**	-0.0490	-0.0477	-0.0448	-0.0415	-0.0375	-0.0327	-0.0269	-0.0217
**UCI**	-0.0294	-0.0259	-0.0209	-0.0161	-0.0113	-0.00629	-0.00123	0.00376
**SE**	0.00497	0.00549	0.00603	0.00642	0.00662	0.00667	0.00648	0.00643
**Constant**	3.299***	3.296***	3.315***	3.322***	3.326***	3.325***	3.321***	3.296***
**LCI**	2.526	2.568	2.627	2.667	2.697	2.718	2.731	2.716
**UCI**	4.072	4.023	4.003	3.978	3.954	3.932	3.910	3.876
**SE**	0.391	0.368	0.348	0.331	0.318	0.307	0.298	0.293
**Observations**	135	135	135	135	135	135	135	135

*** $p < 0.01$, ** $p < 0.05$, * $p < 0.10$

Table S. Global interrupted time-series results with one intervention point, including China. Results presented for 0 to 15-day lags.

	0-day lag	1-day lag	2-day lag	3-day lag	4-day lag	5-day lag	6-day lag	7-day lag
**Time**	-0.0123	-0.0112	-0.0104	-0.00982	-0.00928	-0.00870	-0.00808	-0.00759
**LCI**	-0.0294	-0.0277	-0.0264	-0.0253	-0.0243	-0.0233	-0.0222	-0.0213
**UCI**	0.00482	0.00537	0.00565	0.00567	0.00572	0.00586	0.00607	0.00616
**SE**	0.00864	0.00837	0.00810	0.00783	0.00758	0.00736	0.00715	0.00695
**Feb 5 level change**	0.0596	-0.00236	-0.0524	-0.0875	-0.125	-0.170	-0.221	-0.267
**LCI**	-0.368	-0.416	-0.452	-0.474	-0.500	-0.532	-0.570	-0.603
**UCI**	0.487	0.411	0.347	0.299	0.249	0.192	0.128	0.0696
**SE**	0.216	0.209	0.202	0.195	0.189	0.183	0.177	0.170
**Feb 5 slope change**	0.00730	0.00676	0.00642	0.00627	0.00618	0.00614	0.00618	0.00633
**LCI**	-0.00994	-0.00995	-0.00975	-0.00938	-0.00898	-0.00856	-0.00809	-0.00750
**UCI**	0.0245	0.0235	0.0226	0.0219	0.0213	0.0208	0.0204	0.0202
**SE**	0.00872	0.00844	0.00817	0.00791	0.00766	0.00743	0.00721	0.00699
**Constant**	1.944***	1.924***	1.908***	1.898***	1.888***	1.876***	1.864***	1.854***
**LCI**	1.309	1.293	1.283	1.279	1.274	1.268	1.261	1.256
**UCI**	2.578	2.554	2.534	2.517	2.501	2.484	2.467	2.452
**SE**	0.321	0.319	0.316	0.313	0.310	0.307	0.305	0.302
**Observations**	135	135	135	135	135	135	135	135
	**8-day lag**	**9-day lag**	**10-day lag**	**11-day lag**	**12-day lag**	**13-day lag**	**14-day lag**	**15-day lag**
**Time**	-0.00745	-0.00755	-0.00771	-0.00781	-0.00784	-0.00790	-0.00800	-0.00820
**LCI**	-0.0208	-0.0205	-0.0203	-0.0200	-0.0197	-0.0195	-0.0192	-0.0191
**UCI**	0.00589	0.00539	0.00486	0.00440	0.00403	0.00365	0.00324	0.00274
**SE**	0.00674	0.00654	0.00635	0.00617	0.00600	0.00584	0.00568	0.00553
**Feb 5 level change**	-0.287*	-0.288*	-0.283*	-0.284*	-0.290*	-0.294**	-0.294**	-0.283**
**LCI**	-0.613	-0.606	-0.596	-0.589	-0.588	-0.585	-0.578	-0.562
**UCI**	0.0395	0.0309	0.0286	0.0216	0.00787	-0.00335	-0.00943	-0.00340
**SE**	0.165	0.161	0.158	0.154	0.151	0.147	0.144	0.141
**Feb 5 slope change**	0.00663	0.00700	0.00738	0.00775	0.00814	0.00853	0.00894	0.00932*
**LCI**	-0.00677	-0.00601	-0.00527	-0.00454	-0.00381	-0.00308	-0.00236	-0.00168
**UCI**	0.0200	0.0200	0.0200	0.0200	0.0201	0.0201	0.0202	0.0203
**SE**	0.00678	0.00658	0.00639	0.00621	0.00604	0.00587	0.00571	0.00556
**Constant**	1.851***	1.853***	1.856***	1.859***	1.859***	1.861***	1.863***	1.867***
**LCI**	1.259	1.269	1.279	1.288	1.295	1.303	1.311	1.322
**UCI**	2.443	2.438	2.434	2.429	2.423	2.418	2.414	2.413
**SE**	0.299	0.295	0.292	0.288	0.285	0.282	0.279	0.276
**Observations**	135	135	135	135	135	135	135	135

*** $p < 0.01$, ** $p < 0.05$, * $p < 0.10$

Table T. Result summaries for country-level analyses. Five-day lagged interrupted time-series results for targeted border closures, total border closures, reopening of border closures, and all border closures for every intervention meeting evaluation criteria. Summary ITS outcomes (neutral, beneficial, adverse, or mixed) are presented at the top of the table, and detailed ITS outcomes are summarized in the bottom of the table.

	**Targeted**		**Total**		**Opening**		**All**	
**ITS outcome**	**N**	**%**	**N**	**%**	**N**	**%**	**N**	**%**
**Neutral**	10	29.4%	32	31.1%	3	60.0%	45	31.7%
**Beneficial**	12	35.3%	41	39.8%	0	0.0%	53	37.3%
**Adverse**	10	29.4%	28	27.2%	2	40.0%	40	28.2%
**Mixed**	2	5.9%	2	1.9%	0	0.0%	4	2.8%
**No effect**	10	29.4%	32	31.1%	3	60.0%	45	31.7%
**Level increase, no slope change**	2	5.9%	2	1.9%	1	20.0%	5	3.5%
**Level decrease, no slope change**	9	26.5%	23	22.3%	0	0.0%	32	22.5%
**No level change, slope increase**	5	14.7%	15	14.6%	0	0.0%	20	14.1%
**No level change, slope decrease**	2	5.9%	4	3.9%	0	0.0%	6	4.2%
**Level and slope increase**	3	8.8%	11	10.7%	1	20.0%	15	10.6%
**Level and slope decrease**	1	2.9%	14	13.6%	0	0.0%	15	10.6%
**Level decrease, slope increase**	1	2.9%	1	1.0%	0	0.0%	2	1.4%
**Level increase, slope decrease**	1	2.9%	1	1.0%	0	0.0%	2	1.4%

Table U. Result summaries for country-level falsification tests. Five-day lagged and unlagged interrupted time-series results are presented for countries with total border closures only.

	Lagged				Unlagged			
	Real		Falsification		Real		Falsification	
ITS outcome	N	%	N	%	N	%	N	%
Neutral	11	24.4%	16	25.8%	10	26.3%	15	24.2%
Good	19	42.2%	5	8.1%	21	55.3%	5	8.1%
Bad	14	31.1%	38	61.3%	6	15.8%	40	64.5%
Mixed	1	2.2%	3	4.8%	1	2.6%	2	3.2%
No effect	11	24.4%	16	25.8%	10	26.3%	15	24.2%
Level increase, no slope change	0	0.0%	8	12.9%	2	5.3%	6	9.7%
Level decrease, no slope change	9	20.0%	2	3.2%	8	21.1%	2	3.2%
No level change, slope increase	9	20.0%	12	19.4%	3	7.9%	15	24.2%
No level change, slope decrease	3	6.7%	3	4.8%	3	7.9%	2	3.2%
Level and slope increase	5	11.1%	18	29.0%	1	2.6%	19	30.6%
Level and slope decrease	7	15.6%	0	0.0%	10	26.3%	1	1.6%
Level decrease, slope increase	1	2.2%	2	3.2%	1	2.6%	1	1.6%
Level increase, slope decrease	0	0.0%	1	1.6%	0	0.0%	1	1.6%

Table V. Meta-regression of country-level ITS results. Results of ordered logistic meta-regression of factors associated with the odds of a targeted, total, or any border closures having a positive effect for five-day lagged interrupted time-series results.

	**Single variables**						**Full model**		
	**Targeted**	**Total**			**All**		**Targeted**	**Total**	**All**
**Health expenditures**<br>**SE**	29.09**<br>12.26						29.09**<br>12.26		
**Log of population**<br>**SE**		-0.286**<br>0.121			-0.228**<br>0.103			-0.250*<br>0.131	-0.248**<br>0.115
**GHSI index**<br>**SE**			-0.030**<br>0.0147					-0.0129<br>0.0162	
**First tercile of all restrictions**				omitted		omitted		omitted	omitted
**Second tercile of all restrictions**<br>**SE**				0.218<br>0.458		0.171<br>0.450		0.153<br>0.487	0.303<br>0.455
**Third tercile of all restrictions**<br>**SE**				1.482***<br>0.457		0.980**<br>0.424		1.334***<br>0.493	0.950**<br>0.428
**Observations**	29	89	100	103	119	108	29	85	93

Table W. Meta-regression results for unlagged R_t outcomes. Results for factors associated with the odds of a targeted, total, or any border closures having a positive effect for unlagged results.

	Single variables							
	Targeted		Total				All	
GDP per capita	3.87e-05**							
SE	1.92e-05							
Health expenditures		25.91**						
SE		12.93						
Log of GDP			-0.244**					
SE			0.113					
Log of population				-0.344**			-0.339***	
SE				0.134			0.116	
GHSI score					-0.0448**			
SE					0.0177			
First tercile of all restrictions						(omitted)		(omitted)
Second tercile of all restrictions						0.00889		-0.0182
SE						0.509		0.506
Third tercile of all restrictions						1.315***		1.222***
SE						0.492		0.472
Observations	25	25	87	89	86	89	104	93

	Full model		
	Targeted	Total	All
GDP per capita	4.40e-05**		
SE	1.77e-05		
Health expenditures	32.21**		
SE	14.57		
Log of GDP		0.440*	
SE		0.264	
Log of population		-0.584**	-0.365***
SE		0.234	0.134
GHSI score		-0.0694**	
SE		0.0287	
First tercile of all restrictions		(omitted)	(omitted)
Second tercile of all restrictions		0.0952	-0.0243
SE		0.560	0.519
Third tercile of all restrictions		1.368**	1.141**
SE		0.570	0.481
Observations	24	85	93

Table X. CEM robustness check using higher causal plausibility variables, higher coarsened. Maximum likelihood random-effects estimation for targeted border closures. Country-days are matched on higher coarsened GHSI score, logged GDP, gender parity score, emigrants per capita, democracy index, logged airline passengers, health expenditures, and proportion of female government ministers.

	Targeted border closures			
	**Dropping countries with total border closures**	**Controlling for countries with total border closures**	**Model using % of global population targeted**	**Model using % of global cases targeted**
**Targeted closure (β)**<br>**Confidence interval**	-0.69*** (0.09)<br>[-0.87, -0.50]	-0.55*** (0.06)<br>[-0.68, -0.42]		
**Total closure (β)**<br>**Confidence interval**		-1.29*** (0.05)<br>[-1.39, 1.19]		
**% of global pop targeted (β)**<br>**Confidence interval**			-1.05*** (0.13)<br>[-1.31, 0.80]	
**% of global cases targeted (β)**<br>**Confidence interval**				-0.52*** (0.13)<br>[-0.77, -0.27]
**Constant**<br>**Confidence interval**	2.44*** (0.09)<br>[2.26, 2.61]	2.48*** (0.07)<br>[2.35, 2.61]	2.31*** (0.08)<br>[2.16, 2.46]	2.31*** (0.09)<br>[2.13, 2.48]
**Observations**	2,038	4,790	2,038	4,790
**Countries**	49	49	49	49
	**Total border closures**			
	**Primary Model**		**Conservative Model**	
	**Dropping countries with targeted border closures**	**Controlling for countries with targeted border closures**	**Dropping countries with targeted border closures**	**Controlling for countries with targeted border closures**
**Targeted closure (β)**<br>**Confidence interval**	-0.96*** (0.06)<br>[-1.08, -0.84]	-0.54*** (0.07)<br>[-0.67, -0.40]		-0.48*** (0.11)<br>[-0.69, -0.26]
**Total closure (β)**<br>**Confidence interval**		-1.15*** (0.07)<br>[-1.27, -1.03]	-0.95*** (0.06)<br>[-1.06, -0.84]	-1.00*** (0.08)<br>[-1.15, -0.86]
**Constant**<br>**Confidence interval**	2.13*** (0.07)<br>[1.98, 2.27]	2.17*** (0.09)<br>[2.00, 2.34]	2.31*** (0.06)<br>[2.19, 2.43]	2.49*** (0.09)<br>[2.30, 2.67]
**Observations**	1,445	2,082	2,130	2,902
**Countries**	19	20	35	45

*** $p < 0.01$, ** $p < 0.05$, * $p < 0.10$; standard errors in parentheses

Table Y. CEM robustness check using higher causal plausibility variables, minimally coarsened. Maximum likelihood random-effects estimation for targeted border closures. Country-days are matched on minimally coarsened GHSI score, logged GDP, gender parity score, emigrants per capita, democracy index, logged airline passengers, health expenditures, and proportion of female government ministers.

	**Targeted border closures**			
	**Dropping countries with total border closures**	**Controlling for countries with total border closures**	**Model using % of global population targeted**	**Model using % of global cases targeted**
**Targeted closure (β)**<br>**Confidence interval**	-0.67*** (0.05)<br>[-0.78, -0.57]	-0.70*** (0.04)<br>[-0.78, -0.63]		
**Total closure (β)**<br>**Confidence interval**		-1.25*** (0.03)<br>[-1.31, -1.20]		
**% of global pop targeted (β)**<br>**Confidence interval**			-1.27*** (0.09)<br>[-1.43, -1.10]	
**% of global cases targeted (β)**<br>**Confidence interval**				-0.32*** (0.07)<br>[-0.45, -0.19]
**Constant**<br>**Confidence interval**	2.32*** (0.04)<br>[2.23, 2.41]	2.50*** (0.04)<br>[2.43, 2.58]	2.22*** (0.04)<br>[2.15, 2.30]	2.15*** (0.04)<br>[2.06, 2.23]
**Observations**	5,678	13,545	5,678	5,678
**Countries**	140	140	140	140
	**Total border closures**			
	**Primary Model**		**Conservative Model**	
	**Dropping countries with targeted border closures**	**Controlling for countries with targeted border closures**	**Dropping countries with targeted border closures**	**Controlling for countries with targeted border closures**
**Targeted closure (β)**<br>**Confidence interval**		-0.58*** (0.04)<br>[-0.66, -0.50]		-0.66*** (0.05)<br>[-0.76, -0.56]
**Total closure (β)**<br>**Confidence interval**	-1.07*** (0.03)<br>[-1.12, -1.02]	-1.13*** (0.03)<br>[-1.19, -1.08]	-0.93*** (0.04)<br>[-1.01, -0.86]	-0.96*** (0.04)<br>[-1.04, -0.88]
**Constant**<br>**Confidence interval**	2.30*** (0.03)<br>[2.23, 2.36]	2.32*** (0.04)<br>[2.25, 2.39]	2.28*** (0.04)<br>[2.21, 2.36]	2.34*** (0.04)<br>[2.26, 2.41]
**Observations**	9,455	11,866	7,218	9,817
**Countries**	122	123	129	147

*** p < 0.01, ** p < 0.05, * p < 0.10; standard errors in parentheses

Table Z. CEM robustness check using lower priority variables, minimally coarsened. Maximum likelihood random-effects estimation for targeted border closures. Country-days are matched on minimally coarsened GHSI score, logged GDP, gender parity score, emigrants per capita, and the democracy index.

	**Targeted border closures**			
	**Dropping countries with total border closures**	**Controlling for countries with total border closures**	**Model using % of global population targeted**	**Model using % of global cases targeted**
**Targeted closure (β)**<br>**Confidence interval**	-0.64*** (0.05)<br>[-0.74, -0.53]	-0.68*** (0.04)<br>[-0.75, -0.60]		
**Total closure (β)**<br>**Confidence interval**		-1.19*** (0.03)<br>[-1.25, -1.13]		
**% of global pop targeted (β)**<br>**Confidence interval**			-1.25*** (0.09)<br>[-1.42, -1.07]	
**% of global cases targeted (β)**<br>**Confidence interval**				-0.29*** (0.07)<br>[-0.54, -0.16]
**Constant**<br>**Confidence interval**	2.30*** (0.04)<br>[2.22, 2.39]	2.51*** (0.04)<br>[2.43, 2.58]	2.21*** (0.04)<br>[2.13, 2.28]	2.12*** (0.04)<br>[2.04, 2.21]
**Observations**	5,887	14,440	5,887	5,887
**Countries**	149	154	149	149

	**Total border closures**			
	**Primary Model**		**Conservative Model**	
	**Dropping countries with targeted border closures**	**Controlling for countries with targeted border closures**	**Dropping countries with targeted border closures**	**Controlling for countries with targeted border closures**
**Targeted closure (β)**<br>**Confidence interval**		-0.63*** (0.04)<br>[-0.71, -0.56]		-0.70*** (0.05)<br>[-0.79, -0.61]
**Total closure (β)**<br>**Confidence interval**	-1.00*** (0.03)<br>[-1.06, 0.95]	-1.06*** (0.03)<br>[-1.11, -1.00]	-0.94*** (0.04)<br>[-1.01, -0.87]	-0.97*** (0.04)<br>[-1.04, -0.90]
**Constant**<br>**Confidence interval**	2.33*** (0.03)<br>[2.27, 2.40]	2.35*** (0.03)<br>[2.29, 2.42]	2.29*** (0.04)<br>[2.22, 2.36]	2.37*** (0.04)<br>[2.29, 2.45]
**Observations**	11,581	14,233	7,459	10,223
**Countries**	152	153	136	158

*** $p < 0.01$, ** $p < 0.05$, * $p < 0.10$; standard errors in parentheses

Table AA. CEM robustness check censoring for 45 days only, before and after the two intervention dates, using higher causal plausibility variables, moderately coarsened. Maximum likelihood random-effects estimation for targeted border closures. Country-days are matched on moderately coarsened higher causal plausibility variables, with a reduced time period of 45 days, before and after the global intervention dates for targeted (February 5, 2020) and total border closures (March 19, 2020).

	Targeted border closures			
	Dropping countries with total border closures	Controlling for countries with total border closures	Model using % of global population targeted	Model using % of global cases targeted
**Targeted closure (β)**<br>**Confidence interval**	-0.22*** (0.08)<br>[-0.37, -0.06]	-0.28*** (0.07)<br>[-0.42, -0.14]		
**Total closure (β)**<br>**Confidence interval**		-0.74*** (0.10)<br>[-0.93, -0.56]		
**% of global pop targeted (β)**<br>**Confidence interval**			-0.86*** (0.26)<br>[-1.36, -0.35]	
**% of global cases targeted (β)**<br>**Confidence interval**				0.00 (0.10)<br>[-0.20, -0.19]
**Constant**<br>**Confidence interval**	2.52*** (0.05)<br>[2.43, 2.62]	2.53*** (0.05)<br>[2.44, 2.61]	2.51*** (0.05)	
**Observations**	2,921	3,303	2,921	2,921
**Countries**	149	149	149	149
	**Total border closures**			
	**Primary Model**		**Conservative Model**	
	**Dropping countries with targeted border closures**	**Controlling for countries with targeted border closures**	**Dropping countries with targeted border closures**	**Controlling for countries with targeted border closures**
**Targeted closure (β)**<br>**Confidence interval**		-0.53*** (0.08)<br>[-0.69, -0.36]		-0.59*** (0.06)<br>[-0.71, -0.47]
**Total closure (β)**<br>**Confidence interval**	-1.42*** (0.05)<br>[-1.52, -1.32]	-1.45*** (0.07)<br>[-1.58, -1.32]	-1.06*** (0.06)<br>[-1.18, -0.95]	-1.06*** (0.04)<br>[-1.15, -0.98]
**Constant**<br>**Confidence interval**	2.43*** (0.07)<br>[2.30, 2.56]	2.55*** (0.09)<br>[2.38, 2.73]	2.27*** (0.05)<br>[2.18, 2.36]	2.48*** (0.05)<br>[2.38, 2.59]
**Observations**	2,832	3,547	2,768	6,182
**Countries**	87	78	123	153

*** $p < 0.01$, ** $p < 0.05$, * $p < 0.10$; standard errors in parentheses

Table AB. CEM robustness check censoring for 60 days only, before and after the two intervention dates, using higher causal plausibility variables, moderately coarsened. Maximum likelihood random-effects estimation for targeted border closures. Country-days are matched on moderately coarsened higher causal plausibility variables, with a reduced time period of 60 days, before and after the global intervention dates for targeted (February 5, 2020) and total border closures (March 19, 2020).

	**Targeted border closures**			
	**Dropping countries with total border closures**	**Controlling for countries with total border closures**	**Model using % of global population targeted**	**Model using % of global cases targeted**
**Targeted closure (β)**<br>**Confidence interval**	- 0.38*** (0.07)<br>[-0.51, -0.25]	-0.45*** (0.05)<br>[-0.56, -0.34]		
**Total closure (β)**<br>**Confidence interval**		-1.00*** (0.05)<br>[-1.10, -0.90]		
**% of global pop targeted (β)**<br>**Confidence interval**			-1.14*** (0.15)<br>[-1.42, -0.85]	
**% of global cases targeted (β)**<br>**Confidence interval**				-0.02 (0.09)<br>[-0.20, 0.15]
**Constant**<br>**Confidence interval**	2.39*** (0.05)<br>[2.30, 2.48]	2.49*** (0.04)<br>[2.42, 2.57]	2.37*** (0.04)<br>[2.28, 2.45]	2.28*** (0.05)<br>[2.19, 2.37]
**Observations**	3,487	5,308	3,487	3,487
**Countries**	149	149	149	149
	**Total border closures**			
	**Primary Model**		**Conservative Model**	
	**Dropping countries with targeted border closures**	**Controlling for countries with targeted border closures**	**Dropping countries with targeted border closures**	**Controlling for countries with targeted border closures**
**Targeted closure (β)**<br>**Confidence interval**		-0.45*** (0.10)<br>[-0.64, -0.26]		-0.56*** (0.06)<br>[-0.67, -0.44]
**Total closure (β)**<br>**Confidence interval**	-1.23*** (0.08)<br>[1.37, -1.08]	-1.32*** (0.08)<br>[-1.47, -1.16]	-1.01*** (0.07)<br>[-1.16, -0.87]	-1.05*** (0.05)<br>[-1.14, -0.96]
**Constant**<br>**Confidence interval**	2.27*** (0.07)<br>[2.13, 2.40]	2.42*** (0.08)<br>[2.27, 2.58]	2.26*** (0.05)<br>[2.17, 2.35]	2.43*** (0.05)<br>[2.33, 2.53]
**Observations**	2,829	4,333	3,216	7,506
**Countries**	78	78	153	153

*** p < 0.01, ** p < 0.05, * p < 0.10; standard errors in parentheses

Table AC. CEM robustness check on targeted border closures, using scenario analyses by splitting above and below the mean covariate distributions. Results are presented for maximum likelihood random-effects estimation based on the proportion of global population targeted and matched on different combination of variables. In looking at the effect of targeted border closures, coefficients, standard errors, and confidence intervals are presented.

**Above average**	**Matched on GHSI score, logged GDP, democracy, and proportion of female ministers**		
	**Targeted border closures**	**Model using % of global population targeted**	**Model using % of global cases targeted**
**Targeted closure (β)**<br>**Confidence interval**	-0.88*** (0.11)<br>[-1.10, -0.67]		
**% of global pop targeted (β)**<br>**Confidence interval**		-0.36* (0.19)<br>[-0.73, 0.02]	
**% of global cases targeted (β)**<br>**Confidence interval**			-1.18*** (0.25)<br>[-1.67, -0.69]
**Constant**<br>**Confidence interval**	2.45*** (0.10)<br>[2.25, 2.65]	2.06*** (0.13)<br>[1.81, 2.31]	2.28*** (0.14)<br>[2.01, 2.55]
**Observations**	569	569	569
**Countries**	16	16	16
**Below average**	**Matched on GHSI score, logged GDP, democracy, and proportion of female ministers**		
	**Targeted border closures**	**Model using % of global population targeted**	**Model using % of global cases targeted**
**Targeted closure (β)**<br>**Confidence interval**	-1.26*** (0.11)<br>[-1.47, -1.04]		
**% of global pop targeted (β)**<br>**Confidence interval**		-1.67*** (0.13)<br>[-1.93, -1.42]	
**% of global cases targeted (β)**<br>**Confidence interval**			-0.42*** (0.124)<br>[-0.66, -0.17]
**Constant**<br>**Confidence interval**	2.69*** (0.13)<br>[2.44, 2.94]	2.44*** (0.10)<br>[2.24, 2.64]	2.21*** (0.11)<br>[1.99, 2.43]
**Observations**	1,588	1,588	1,588
**Countries**	24	24	24
**Above average**	**Matched on GHSI score, logged GDP, and democracy**		
	**Targeted border closures**	**Model using % of global population targeted**	**Model using % of global cases targeted**
**Targeted closure (β)**<br>**Confidence interval**	-0.69*** (0.14)<br>[-0.97, -0.42]		
**% of global pop targeted (β)**<br>**Confidence interval**		-0.58* (0.31)<br>[-1.18, 0.02]	
**% of global cases targeted (β)**<br>**Confidence interval**			-0.79*** (0.29)<br>[-1.36, -0.22]
**Constant**<br>**Confidence interval**	2.21*** (0.10)<br>[2.01, 2.41]	1.98*** (0.11)<br>[1.77, 2.19]	2.07*** (0.12)<br>[1.83, 2.30]
**Observations**	781	781	781
**Countries**	22	22	22
**Below average**	**Matched on GHSI score, logged GDP, and democracy**		
	**Targeted border closures**	**Model using % of global population targeted**	**Model using % of global cases targeted**
**Targeted closure (β)**<br>**Confidence interval**	-1.11*** (0.09)<br>[-1.28, -0.94]		
**% of global pop targeted (β)**<br>**Confidence interval**		-1.67*** (0.11)<br>[-1.88, -1.46]	
**% of global cases targeted (β)**<br>**Confidence interval**			-0.27*** (0.10)<br>[-0.46, -0.07]
**Constant**<br>**Confidence interval**	2.67*** (0.10)<br>[2.47, 2.87]	2.44*** (0.08)<br>[2.29, 2.60]	2.17*** (0.10)<br>[1.97, 2.36]

**Observations**	2,224	2,224	2,224
**Countries**	34	34	34
**Above average**	**Matched on GHSI score and democracy**		
	**Targeted border closures**	**Model using % of global population targeted**	**Model using % of global cases targeted**
**Targeted closure (β)**<br>**Confidence interval**	-0.38** (0.16)<br>[-0.69, -0.06]		
**% of global pop targeted (β)**<br>**Confidence interval**		-0.58** (0.27)<br>[-1.10, -0.05]	
**% of global cases targeted (β)**<br>**Confidence interval**			-0.18 (0.24)<br>[-0.66, 0.30]
**Constant**<br>**Confidence interval**	2.22*** (0.13)<br>[1.97, 2.47]	2.11*** (0.10)<br>[1.91, 2.32]	2.09*** (0.12)<br>[1.85, 2.32]
**Observations**	1,197	1,197	1,197
**Countries**	35	35	35
**Below average**	**Matched on GHSI score and democracy**		
	**Targeted border closures**	**Model using % of global population targeted**	**Model using % of global cases targeted**
**Targeted closure (β)**<br>**Confidence interval**	-1.11*** (0.08)<br>[-1.27, -0.95]		
**% of global pop targeted (β)**<br>**Confidence interval**		-1.71*** (0.11)<br>[-1.92, -1.50]	
**% of global cases targeted (β)**<br>**Confidence interval**			-0.38*** (0.09)<br>[-0.57, -0.20]
**Constant**<br>**Confidence interval**	2.72*** (0.09)<br>[2.54, 2.90]	2.47*** (0.08)<br>[2.32, 2.62]	2.27*** (0.10)<br>[2.09, 2.44]
**Observations**	2,458	2,458	2,458
**Countries**	44	44	44

***p<0.01, **p<0.05, *p<0.1; standard errors in parentheses

Table AD. CEM robustness check on total border closures, using scenario analyses with covariate distributions split by above average and below average. Results are presented for maximum likelihood random-effects estimation based on the proportion of global population targeted.

	Matched on GHSI score, logged GDP, democracy, and proportion of female ministers	
	**Below average**	**Above average**
**Total closure (β)**<br>**Confidence interval**	-0.84*** (0.10)<br>[-1.03, -0.65]	-1.65*** (0.06)<br>[-1.76, -1.54]
**Constant**<br>**Confidence interval**	2.39*** (0.10)<br>[2.19, 2.60]	2.56*** (0.10)<br>[2.38, 2.74]
**Observations**	1,146	1,828
**Countries**	17	24
	**Matched on GHSI score, logged GDP, and democracy**	
	**Below average**	**Above average**
**Total closure (β)**<br>**Confidence interval**	-0.67*** (0.11)<br>[-0.88, -0.46]	-1.59*** (0.05)<br>[-1.69, 1.50]
**Constant**<br>**Confidence interval**	2.23*** (0.10)<br>[2.03, 2.42]	2.54*** (0.07)<br>[2.40, 2.68]
**Observations**	1,764	2,418
**Countries**	25	34
	**Matched on GHSI score and democracy**	
	**Below average**	**Above average**
**Total closure (β)**<br>**Confidence interval**	-0.73*** (0.08)<br>[-0.89, -0.58]	-1.50*** (0.05)<br>[-1.59, -1.41]
**Constant**<br>**Confidence interval**	2.22*** (0.08)<br>[2.07, 2.37]	2.54*** (0.06)<br>[2.42, 2.66]
**Observations**	2,696	3,471
**Countries**	36	46

***p<0.01, **p<0.05, *p<0.1; standard errors in parentheses

Table AE. CEM robustness check using containment and closure policy variables, moderately coarsened. Maximum likelihood random-effects estimation for targeted border closures. Country-days are matched on moderately coarsened containment and closure policy changes, such as closures on workplaces and public transit, stay at home orders, restrictions on public gatherings and on internal movement.

	**Targeted border closures**			
	**Dropping countries with total border closures**	**Controlling for countries with total border closures**	**Model using % of global population targeted**	**Model using % of global cases targeted**
**Targeted closure (β)**<br>**Confidence interval**	-0.53*** (0.05)<br>[-0.63, -0.42]	-0.64*** (0.04)<br>[-0.72, -0.56]		
**Total closure (β)**<br>**Confidence interval**		-1.15*** (0.04)<br>[-1.22, -1.08]		
**% of global pop targeted (β)**<br>**Confidence interval**			-1.12*** (0.09)<br>[-1.30, -0.94]	
**% of global cases targeted (β)**<br>**Confidence interval**				-0.18*** (0.07)<br>[-0.31, -0.04]
**Constant**<br>**Confidence interval**	2.06*** (0.04)<br>[1.98, 2.14]	2.49*** (0.04)<br>[2.41, 2.56]	2.02*** (0.04)<br>[1.94, 2.09]	1.95*** (0.04)<br>[1.88, 2.03]
**Observations**	5,842	14,806	5,842	5,842
**Countries**	161	161	161	161
	**Total border closures**			
	**Primary Model**		**Conservative Model**	
	**Dropping countries with targeted border closures**	**Controlling for countries with targeted border closures**	**Dropping countries with targeted border closures**	**Controlling for countries with targeted border closures**
**Targeted closure (β)**<br>**Confidence interval**	-0.99*** (0.03)<br>[-1.04, -0.94]	-0.43*** (0.04)<br>[-0.50, -0.35]		-0.34*** (0.05)<br>[-0.44, -0.25]
**Total closure (β)**<br>**Confidence interval**		-0.99*** (0.03)<br>[-1.04, -0.94]	-0.91*** (0.03)<br>[-0.98, -0.85]	-0.89*** (0.03)<br>[-0.96, -0.83]
**Constant**<br>**Confidence interval**	2.18*** (0.04)<br>[2.10, 2.25]	2.17*** (0.04)<br>[2.09, 2.25]	1.71*** (0.04)<br>[1.64, 1.78]	1.95*** (0.04)<br>[1.86, 2.03]
**Observations**	11,110	14,503	6,936	10,298
**Countries**	166	166	166	166

*** $p < 0.01$, ** $p < 0.05$, * $p < 0.10$; standard errors in parentheses

Table AF. CEM robustness check using containment and closure variables, restricting dataset to countries originally matched on, moderately coarsened. Maximum likelihood random-effects estimation for targeted border closures. Country-days are matched on moderately coarsened containment and closure policy changes, restricting only to the countries that were matched upon in the primary analysis. Variables being matched on include closures on workplaces and public transit, stay at home orders, restrictions on public gatherings and on internal movement.

	Targeted border closures			
	**Dropping countries with total border closures**	**Controlling for countries with total border closures**	**Model using % of global population targeted**	**Model using % of global cases targeted**
**Targeted closure (β)**<br>**Confidence interval**	-0.60*** (0.06)<br>[-0.71, -0.48]	-0.59*** (0.04)<br>[-0.68, -0.51]		
**Total closure (β)**<br>**Confidence interval**		-1.19*** (0.04)<br>[-1.27, -1.11]		
**% of global pop targeted (β)**<br>**Confidence interval**			-1.15*** (0.09)<br>[-1.33, -0.96]	
**% of global cases targeted (β)**<br>**Confidence interval**				-0.01 (0.07)<br>[-0.15, 0.13]
**Constant**<br>**Confidence interval**	2.02*** (0.05)<br>[1.92, 2.11]	2.46*** (0.04)<br>[2.38, 2.55]	1.97*** (0.05)<br>[1.88, 2.06]	1.89*** (0.05)<br>[1.80, 1.98]
**Observations**	4,384	10,563	4,384	4,384
**Countries**	111	111	111	111

	Total border closures			
	**Primary Model**		**Conservative Model**	
	**Dropping countries with targeted border closures**	**Controlling for countries with targeted border closures**	**Dropping countries with targeted border closures**	**Controlling for countries with targeted border closures**
**Targeted closure (β)**<br>**Confidence interval**		-0.40*** (0.07)<br>[-0.54, -0.26]		-0.18*** (0.06)<br>[-0.31, -0.06]
**Total closure (β)**<br>**Confidence interval**	-1.19*** (0.05)<br>[-1.30, -1.10]	-1.23*** (0.05)<br>[-1.34, -1.13]	-0.88*** (0.04)<br>[-0.96, -0.80]	-0.86*** (0.04)<br>[-0.94, -0.78]
**Constant**<br>**Confidence interval**	1.83*** (0.07)<br>[1.78, 2.08]	1.93*** (0.08)<br>[1.89. 2.09]	1.69*** (0.04)<br>[1.60, 1.77]	1.82*** (0.06)<br>[1.71, 1.93]
**Observations**	3,051	4,633	4,680	7,153
**Countries**	45	45	111	111

*** $p < 0.01$, ** $p < 0.05$, * $p < 0.10$; standard errors in parentheses

Table AG. CEM robustness check using containment and closure variables and higher causal plausibility variables, moderately coarsened. Maximum likelihood random-effects estimation for targeted border closures. Country-days are matched on moderately coarsened containment and closure policy changes and the higher causal plausibility variables, restricting only to the countries that were matched upon in the primary analysis. Variables being matched on include the GHSI score, logged GDP, logged passengers flown, health expenditures, closures on workplaces and public transit, stay at home orders, and restrictions on public gatherings.

	**Targeted border closures**			
	**Dropping countries with total border closures**	**Controlling for countries with total border closures**	**Model using % of global population targeted**	**Model using % of global cases targeted**
**Targeted closure (β)**<br>**Confidence interval**	-0.70*** (0.07)<br>[-0.83, -0.57]	-0.62*** (0.05)<br>[-0.71, -0.53]		
**Total closure (β)**<br>**Confidence interval**		-1.23*** (0.04)<br>[-1.31, -1.15]		
**% of global pop targeted (β)**<br>**Confidence interval**			-1.33*** (0.10)<br>[-1.52, -1.14]	
**% of global cases targeted (β)**<br>**Confidence interval**				-0.08 (0.08)<br>[-0.23, -0.08]
**Constant**<br>**Confidence interval**	2.12*** (0.06)<br>[2.10, 2.33]	2.50*** (0.04)<br>[2.41, 2.58]	2.15*** (0.05)<br>[2.04, 2.26]	2.05*** (0.06)<br>[1.94, 2.16]
**Observations**	3,894	9,865	3,894	3,894
**Countries**	111	111	111	111
	**Total border closures**			
	**Primary Model**		**Conservative Model**	
	**Dropping countries with targeted border closures**	**Controlling for countries with targeted border closures**	**Dropping countries with targeted border closures**	**Controlling for countries with targeted border closures**
**Targeted closure (β)**<br>**Confidence interval**		-0.48*** (0.07)<br>[-0.62, -0.34]		-0.53*** (0.06)<br>[-0.65, -0.41]
**Total closure (β)**<br>**Confidence interval**	-1.37*** (0.07)<br>[-1.51, -1.23]	-1.44*** (0.06)<br>[-1.56, -1.31]	-0.83*** (0.09)<br>[-1.01, -0.66]	-0.97*** (0.05)<br>[-1.07, -0.88]
**Constant**<br>**Confidence interval**	2.23*** (0.11)<br>[2.03, 2.44]	2.44*** (0.10)<br>[2.25, 2.63]	2.32*** (0.05)<br>[2.23, 2.41]	2.34*** (0.05)<br>[2.24, 2.45]
**Observations**	1,704	3,390	2,490	6,595
**Countries**	44	44	110	110

*** p < 0.01, ** p < 0.05, * p < 0.10; standard errors in parentheses

Table AH. Fixed-effects regression analyses. Results are presented for all factors found to produce statistically significant fixed-effects interactions between the extent of border closures and their effect on five-day lagged R_t for the Population Model (based on the proportion of population targeted) and Case Model (based on the proportion of cases targeted). The number of countries, regression coefficient, and direction of effect are shown below.

**Variable**	**N**	**Model using % of global population targeted**		**Model using % of global cases targeted**	
		**Coefficient**	**Direction**	**Coefficient**	**Direction**
**Country**					
Number of airline passengers (logged)	134	-0.093***	Negative		
Emigrants per capita	158	1.067***	Positive		
Participatory democracy index	159	-1.487***	Negative		
Region	164				
Americas (omitted)					
Africa		0.322**	Positive	-1.086***	Negative
Eastern Mediterranean		0.086			
Europe		-0.467***	Negative		
South-East Asia		0.336*	Positive		
Western Pacific		0.502**	Positive	0.642*	Positive
**Economy**					
Income level	164				
Low income (omitted)					
Lower middle income		-0.195		-0.133	
Upper middle income		-0.570***	Negative	-0.533***	Negative
High income		-0.774***	Negative	-0.450*	Negative
GDP (logged)	157	-0.197***	Negative		
**Gender**					
Gender parity rank	159	0.004***	Positive	0.005***	Positive
Gender parity score	159	-0.010***	Negative	-0.014***	Negative
Proportion of women ministers	157	-0.010***	Negative	-0.010**	Negative
Proportion of women legislators	116	-1.220**	Negative	-1.390**	Negative
**Health**					
GHSI score	161	-0.021***	Negative		
GHSI rank	161	0.007***	Positive		
Current health expenditure (% of GDP)	157	-10.210***	Negative		
**Policy control indicators**					
School closure	160	-0.316*	Negative		
Workplace closure	160	-0.831***	Negative	-0.605***	Negative
Restrictions on gatherings	160	-0.894***	Negative	-0.239	
Close public transport	160	-0.822***	Negative	-0.724***	Negative
Stay at home order	160	-0.817***	Negative	-0.858***	Negative
Internal movement restriction	160	-0.891***	Negative	-0.847***	Negative

*** p<0.01, ** p<0.05, *p<0.1

Table AI. All fixed-effects regression analyses. Results are presented for all factors hypothesized to affect the extent of border closures and their effect on R_t, up to 15-day lags. The Population Model is based on the proportion of population targeted and the Case Model is based on the proportion of cases targeted. The number of countries, regression coefficient, and direction of effect are shown below.

Variable	N	Model using % of global population targeted				Model using % of global cases targeted			
		0-day lag	5-day lag	10-day lag	15-day lag	0-day lag	5-day lag	10-day lag	15-day lag
**Country**									
Number of airline passengers (logged)	134	-0.083**	-0.093***	-0.115***	-0.125***	0.042	0.025	-0.006	-0.030
Emigrants per capita	158	0.974**	1.067***	1.079***	1.162***	-0.150	0.164	0.380	0.648
Self-reported trust levels by population in others	57	0.112	-0.003	-0.343	-0.588	0.619	0.619	0.361	-0.064
Population	160	-2.54e-10	-2.92e-10	-4.47e-10	-5.81e-10**	2.33e-10	2.05e-10	1.21e-10	0
Democracy index	159	-1.604***	-1.487***	-1.397***	-1.219***	-0.889	-0.908	-0.915*	-0.809*
**Economy**									
GDP (logged)	157	-0.194***	-0.197***	-0.207***	-0.203***	-0.016	-0.030	-0.051	-0.070
**Gender**									
Parity rank	159	0.003**	0.004***	0.003***	0.003***	0.004**	0.005***	0.004***	0.003***
Parity score	159	-0.010***	-0.010***	-0.010***	-0.009***	-0.014***	-0.014***	-0.013***	-0.011***
Proportion of women ministers	157	-0.010***	-0.010***	-0.009***	-0.008***	-0.009*	-0.010**	-0.010**	-0.008*
Percentage of women in local bodies	156	-0.124	-0.171	-0.185	-0.149	-0.342	-0.389	-0.295	-0.185
Proportion of women legislators	159	0.003**	0.004***	0.003***	0.003***	0.004**	0.005***	0.004***	0.003***
**Health**									
GHSI score	116	-1.048*	-1.220**	-1.262***	-1.052**	-1.237	-1.390**	-1.333**	-1.001*
GHSI index	161	0.007***	0.007***	0.007***	0.007***	0.003	0.003	0.003**	0.003**
Health expenditure	161	-0.022***	-0.021***	-0.023***	-0.022***	-0.006	-0.007	-0.009	-0.010
**Policy control variables**									
School closure	160	0.316*	0.431***	0.384**	0.201	-0.094	0.014	-0.016	-0.09
Workplace closure	160	0.831***	0.768***	0.793***	0.769***	0.605***	0.517***	0.532***	0.547***
Stay at home order	160	0.817***	0.894***	1.993***	0.984***	0.858***	0.878***	0.936***	0.895***
Internal movement restriction	160	0.891***	0.805***	0.854***	0.876***	0.847***	0.714***	0.740***	0.744***

*** p<0.01, ** p<0.05, *p<0.1

Table AJ. Imbalance checks for primary coarsened exact matching analysis. L1 statistics are presented as verification of the improved balance after coarsened exact matching is conducted.

**Intervention**	**Time period**	**L1 statistic (before matching)**	**L1 statistic (after matching)**	**Outcome**
Targeted border closures	Feb 1, 2021	0.14594	0.13554	Improved balance
Targeted border closures	Mar 15, 2021	0.164425	0.13891	Improved balance
Targeted border closures	May 1, 2021	0.14594	0.13354	Improved balance
Total border closures	Feb 1, 2021	0.15787	0.13579	Improved balance
Total border closures	Mar 15, 2021	0.22606	0.13750	Improved balance
Total border closures	May 1, 2021	0.31815	0.16380	Improved balance

Table AK. CEM robustness check using variables from the highest R-squared model, moderately coarsened. Maximum likelihood random-effects estimation for targeted border closures. Country-days are matched on moderately coarsened logged passengers flown, logged GDP, proportions of female ministers, and health expenditure. These variables result in the highest R-squared combination of variables from each main subcategory (country, economy, gender, health, and containment and closure).

	**Targeted border closures**			
	**Dropping countries with total border closures**	**Controlling for countries with total border closures**	**Model using % of global population targeted**	**Model using % of global cases targeted**
**Targeted closure (β)**<br>**Confidence interval**	-0.81*** (0.06)<br>[-0.92, -0.70]	-0.68*** (0.04)<br>[-0.75, -0.61]		
**Total closure (β)**<br>**Confidence interval**		-1.22*** (0.03)<br>[-1.27, -1.17]		
**% of global pop targeted (β)**<br>**Confidence interval**			-1.40*** (0.08)<br>[-1.56, -1.24]	
**% of global cases targeted (β)**<br>**Confidence interval**				-0.38*** (0.07)<br>[-0.52, -0.25]
**Constant**<br>**Confidence interval**	2.52*** (0.05)<br>[2.42, 2.63]	2.48*** (0.04)<br>[2.41, 2.55]	2.38*** (0.05)<br>[2.29, -2.47]	2.28*** (0.05)<br>[2.18, 2.38]
**Observations**	5,059	13,719	5,059	5,059
**Countries**	138	145	138	138
	**Total border closures**			
	**Primary Model**		**Conservative Model**	
	**Dropping countries with targeted border closures**	**Controlling for countries with targeted border closures**	**Dropping countries with targeted border closures**	**Controlling for countries with targeted border closures**
**Targeted closure (β)**<br>**Confidence interval**		-0.67*** (0.05)<br>[-0.76, -0.58]		-0.81*** (0.05)<br>[-0.91, -0.71]
**Total closure (β)**<br>**Confidence interval**	-1.24*** (0.03)<br>[-1.30, -1.18]	-1.34*** (0.04)<br>[-1.41, -1.26]	-1.10*** (0.04)<br>[-1.17, -1.02]	-1.13*** (0.04)<br>[-1.21. -1.05]
**Constant**<br>**Confidence interval**	2.42*** (0.04)<br>[2.35, 2.49]	2.45*** (0.05)<br>[2.36, 2.54]	2.48*** (0.04)<br>[2.40, 2.56]	2.55*** (0.04)<br>[2.47, 2.63]
**Observations**	6,656	8,761	6,700	9,306
**Countries**	83	85	122	144

*** p < 0.01, ** p < 0.05, * p < 0.10; standard errors in parentheses

S1 Data. Matrix of border-closure status by country-day. Travel restriction status by country-day of every border closure included in the study analyses [available as csv on Scholars Portal].

S2 Data. Country-specific interrupted time-series results. Results presented for 0 to 15-day lags [available as csv on Scholars Portal].

S3 Data. Analysis plan. Ex ante analysis plan with changes to analytical approach noted [available as pdf on Scholars Portal].

S4 Data. Global and country-specific interrupted time-series results. Dickey-Fuller tests for unit roots, Cumby-Huizinga tests for autocorrelation, and results correcting for each lag found to have serial correlation present for all global and country interrupted time series [available as csv on Scholars Portal].
