# Supplementary material for: Quasi-experimental evaluation of national border closures on COVID-19 transmission
Source: PLOS Glob Public Health. 2023 Feb 28;3(2):e0000980. doi: 10.1371/journal.pgph.0000980 (PMC10021705; doi:10.1371/journal.pgph.0000980)
Supplement: S3 Data — Ex ante analysis plan with changes to analytical approach noted [available as pdf on Scholars Portal]. (DOCX) [file pgph.0000980.s004.docx]

**Last updated:** December 2021

**Title**

COVID-19 National Border Closure Project Analysis Plan (Quasi-experimental evaluation of national border closures

on COVID-19 transmission)

**Research objective**

To investigate whether and under what conditions targeted and total border closures have affected the transmission of COVID-19 on a global scale.

**Research methodology**

Multi-method approach to triangulate results from interrupted time-series analysis, meta-regression, coarsened exact matching, and an extensive series of robustness checks to evaluate the effect of 166 countries’ national border closures on the global transmissions of COVID-19.

**
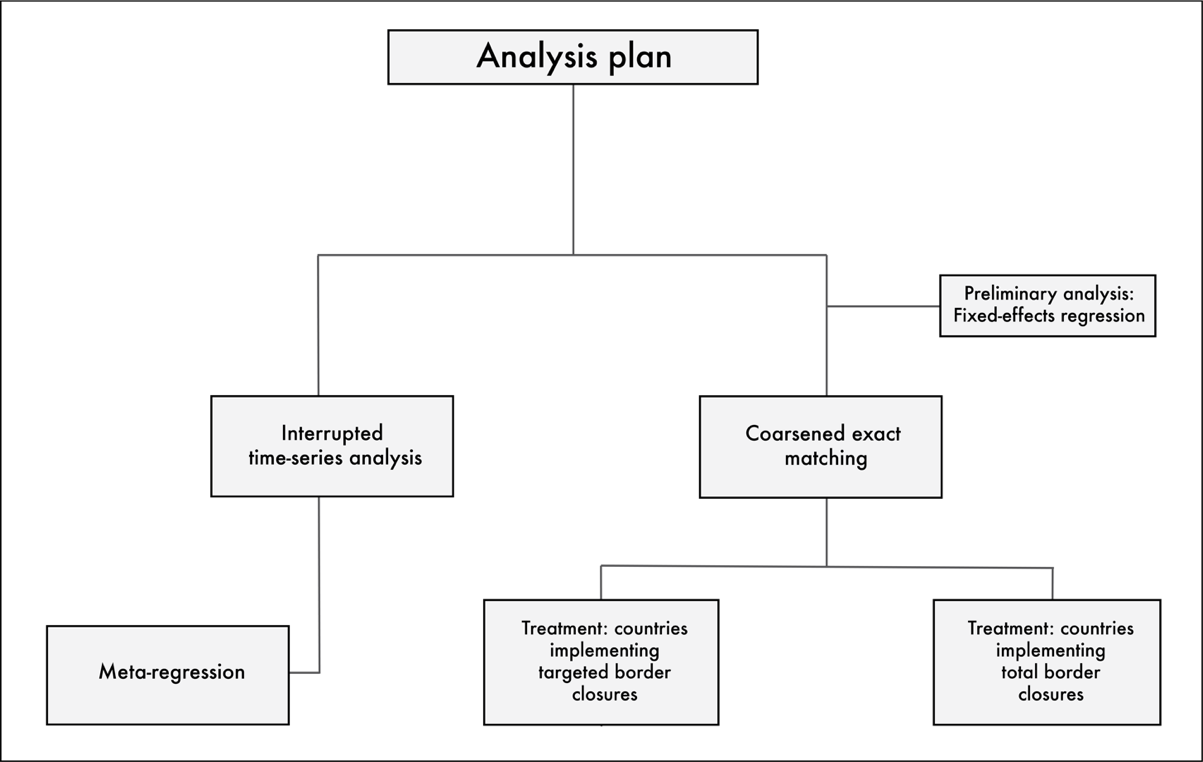
**

Figure 1: Flow diagram of analyses being conducted

**Phase 0: Time series regression analysis**

Using country-level fixed effects, we will evaluate the effect of both targeted and total travel restrictions. This is a first stage of exploratory quantitative analysis that will be used to inform subsequent quantitative methods.

- Covariates found to significantly modify the relationship between travel restrictions and COVID-19 cases and deaths will be carried forward in Phase 2 analyses.
  - Other covariates to be included in analysis include other government imposed COVID-19 interventions as measured by OxCGRT, population density, trade openness.
  - A weakness of current data is that gender-stratified cases and deaths are not currently available. Rather than probing the effects on gender-specific outcomes, we will investigate the effect of the proportion of seats held by women in national parliaments as a factor that may affect whether targeted travel restrictions are put in place.
- Analyses will be repeated using each dependent variable and stratification outlined in the previous section.

**Phase 1A: Matching analysis**

Coarsened Exact Matching (CEM) using covariates found to significantly alter the relationship between travel bans and dependent variables in Phase 1B will be used to explore whether countries matched for similarity by decision of implementing one or multiple states of travel restrictions experienced different COVID-19 outcome trajectories.

- Propensity score matching will not be used because of stronger assumptions needed to use the method and higher risk of bias introduced in the first stage of generating propensity scores than with CEM.
- Weights derived from CEM will be used in a time series regression without country-level fixed effects, resulting in more intuitive results in terms of matching similar countries and calculating the average treatment effect on the treated (ATET).

**Phase 1B: Alignment analysis**

Alignment-based analyses will be used to evaluate the impact of targeted and complete travel restrictions at the country level and by pooling groups of countries.

- Interrupted time series (ITS) analysis will be run for every country with complete data, resulting in a measure of slope change and level change for both targeted and complete travel bans.
  - Some countries will require a multi-stage interruption design and escalation and de-escalation will be evaluated as separate interventions.
  - We will investigate the possibility of incorporating time-varying covariates such as other government imposed COVID-19 control measures.
- A matched ITS with case countries (implementers) and control countries (non-implementers) will be aligned at the time of implementation (t=0) and evaluated for changes in both slope and level of outcome variables.
- We will run pooled ITS through different stratifications, including countries implementing early and late travel restrictions (i.e. was the restriction implemented pre- or post-100 case mark), and other factors detailed in the project plan.
- We will conduct meta-regression by ordered logistic regression to further evaluate country-level factors as associated with border closure effects

**Data & materials**

**Primary intervention variable: travel restrictions**

- We will define travel restrictions as a government-imposed legal restriction to international travel implemented at the national level primarily targeted to non-citizens, but which may also affect citizens.
  - Data permitting, we will also evaluate *de facto* travel restrictions, such as state-owned airlines halting all international flights or flights to a specific country or region.
  - *De facto* travel restrictions also include the closing of a land or sea border by a country to a neighbouring country.
- Travel restrictions are primarily divided between total and targeted border closures.
  - Total travel restrictions are total border closures or restrictions on travel of non-citizens applied equally to all regions.
  - Targeted travel restrictions are restrictions on travel of non-citizens applied selectively to specified countries or regions. For the purposes of this project, we do not classify quarantine or increased monitoring targeted to travellers from specified regions as a travel restriction.
- Our primary data source for extracting travel restriction information will be the Oxford COVID-19 Government Response Tracker ([OxCGRT](https://covidtracker.bsg.ox.ac.uk/)).

**Primary outcome variable: COVID-19 outcome measure**

Due to insufficient and extreme variation in testing capacity, asymptomatic transmission, and incomplete accounting of deaths, there is no perfect way to measure the transmission of COVID-19 internationally. We will rely on three ways of quantifying the burden of COVID-19 including: excess mortality, statistically adjusted COVID-19 cases and deaths, and confirmed COVID-19 cases and deaths.

1. Excess mortality will be used as a method of comparing the relative mortality burden between countries. Excess mortality is a more directly comparable outcome for countries where it is available, but not all excess mortality can be attributed to COVID-19.
   1. One source is [The Economist](file:///Volumes/GoogleDrive/.shortcut-targets-by-id/17rHB5kPXG8uPt85RUPZxkTNyhLJgoOXh/COVID%20Travel%20Restrictions/Dataverse%20uploads/i.%09https:/github.com/TheEconomist/covid-19-excess-deaths-tracker), which currently compiles data for 20 countries (mostly high-income, with some middle-income countries available including Ecuador, Indonesia, Turkey).
   2. Another source is [The Financial Times](file:///Volumes/GoogleDrive/.shortcut-targets-by-id/17rHB5kPXG8uPt85RUPZxkTNyhLJgoOXh/COVID%20Travel%20Restrictions/Dataverse%20uploads/i.%09https:/github.com/Financial-Times/coronavirus-excess-mortality-data), which currently compiles data for 24 countries.
2. Reported cases and mortality are regularly compiled by a number of different organizations, but due to varying testing levels and different methods of linking deaths to COVID-19, statistical adjustments to reported cases and deaths provide added international comparability.
   1. We will rely on [IHME](file:///Volumes/GoogleDrive/.shortcut-targets-by-id/17rHB5kPXG8uPt85RUPZxkTNyhLJgoOXh/COVID%20Travel%20Restrictions/Dataverse%20uploads/i.%09https:/covid19.healthdata.org) estimated mortality and cases, which currently has rapidly expanding set of international data, with notable exception of Africa, the Middle East, and much of Asia.
      - We will ensure that modelling is not based on our covariates to ensure that we do not run into problems of endogeneity.
3. As a third option, we will use reported numbers of confirmed cases and deaths, which are the least reliable in terms of international comparability but offer the advantage of more complete global coverage. The primary source is the COVID-19 Dashboard from [The Center for Systems Science and Engineering (CSSE)](file:///Volumes/GoogleDrive/.shortcut-targets-by-id/17rHB5kPXG8uPt85RUPZxkTNyhLJgoOXh/COVID%20Travel%20Restrictions/Dataverse%20uploads/i.%09https:/coronavirus.jhu.edu/map.html) at Johns Hopkins University (JHU).

**Covariates: country characteristics**

| **Variable** | **Descriptor** |
| --- | --- |
| **Country** | |
| Region | Region defined by the WHO |
| Population, [source](https://population.un.org/wpp/Download/Standard/Population/) | Population in 2020 |
| Democracy, [source](file:///Volumes/GoogleDrive/.shortcut-targets-by-id/17rHB5kPXG8uPt85RUPZxkTNyhLJgoOXh/COVID%20Travel%20Restrictions/Dataverse%20uploads/%09https:/www.v-dem.net/en/data/data/v-dem-dataset) | Participatory democracy index provided by V-Dem (version 10) |
| Level of trust in a population, [source](file:///Volumes/GoogleDrive/.shortcut-targets-by-id/17rHB5kPXG8uPt85RUPZxkTNyhLJgoOXh/COVID%20Travel%20Restrictions/Dataverse%20uploads/%09http:/www.worldvaluessurvey.org/wvs.jsp) | The World Values Survey indicator for social cohesion within a country’s borders |
| Migrants per capita, [source](file:////Volumes/GoogleDrive/.shortcut-targets-by-id/17rHB5kPXG8uPt85RUPZxkTNyhLJgoOXh/COVID%20Travel%20Restrictions/Dataverse%20uploads/https:/migrationdataportal.org/data%3fi=stock_abs_origin&t=2019) | UN DESA’s measure of the proportion of citizens abroad (globalized citizenry) |
| Passengers per capita, [source](file:///Volumes/GoogleDrive/.shortcut-targets-by-id/17rHB5kPXG8uPt85RUPZxkTNyhLJgoOXh/COVID%20Travel%20Restrictions/Dataverse%20uploads/•%09https:/data.worldbank.org/indicator/IS.AIR.PSGR) | A World Bank measure of a country’s exposure to international airline traffic |
| **Economy** | |
| Income level, [source](file:///Volumes/GoogleDrive/.shortcut-targets-by-id/17rHB5kPXG8uPt85RUPZxkTNyhLJgoOXh/COVID%20Travel%20Restrictions/Dataverse%20uploads/%09https:/datahelpdesk.worldbank.org/knowledgebase/articles/906519-world-bank-country-and-lending-groups) | World Bank’s income level categories: high, upper-middle, lower-middle, and lower |
| GDP, [source](https://data.worldbank.org/indicator/NY.GDP.MKTP.CD) | Measure of a country’s level of annual gross domestic product |
| **Gender** | |
| Proportion of female seats, government bodies, [source](file:///Volumes/GoogleDrive/.shortcut-targets-by-id/17rHB5kPXG8uPt85RUPZxkTNyhLJgoOXh/COVID%20Travel%20Restrictions/Dataverse%20uploads/%09https:/www.cfr.org/article/womens-power-index) | The Council of Foreign Relation’s measure of the proportion of seats held by women in government positions (i.e.: ministerial, local bodies, national legislature, parity ranking) |
| **Health** | |
| Domestic health spending as a percentage of GDP, [source](file:///Volumes/GoogleDrive/.shortcut-targets-by-id/17rHB5kPXG8uPt85RUPZxkTNyhLJgoOXh/COVID%20Travel%20Restrictions/Dataverse%20uploads/•%09https:/data.worldbank.org/indicator/SH.XPD.CHEX.GD.ZS) | A World Bank measure of domestic health spending |
| Pandemic preparedness measure, [source](file:///Volumes/GoogleDrive/.shortcut-targets-by-id/17rHB5kPXG8uPt85RUPZxkTNyhLJgoOXh/COVID%20Travel%20Restrictions/Dataverse%20uploads/•%09https:/www.ghsindex.org) | GHS Index |
| **Restriction** | |
| Stage of adoption | A measure of domestic case load and date on the extent of early and late adoption of travel restrictions by a country |
