# Supplementary material for: Quasi-experimental evaluation of national border closures on COVID-19 transmission
Source: PLOS Glob Public Health. 2023 Feb 28;3(2):e0000980. doi: 10.1371/journal.pgph.0000980 (PMC10021705; doi:10.1371/journal.pgph.0000980)
Supplement: Attachment — Submitted filename: Response to reviewers (08-05-2022).pdf [file pgph.0000980.s006.pdf]

YORK
UNIVERSITÉ
UNIVERSITY
GLOBAL STRATEGY LAB

Dahdaleh Institute for Global Health Research

DAHDALEH BUILDING, 2120
4700 KEELE ST
TORONTO ON
CANADA M3J 1P3
T 416 736 2100

www.globalstrategylab.org

Response to Reviewers

Journal Requirements

1. Please send a completed 'Competing Interests' statement, including any COIs declared by your co-authors. If you have no competing interests to declare, please state "The authors have declared that no competing interests exist". Otherwise please declare all competing interests beginning with the statement "I have read the journal's policy and the authors of this manuscript have the following competing interests:"

- *Competing interests: SJH is the Vice-President of Corporate Data & Surveillance at the Public Health Agency of Canada (PHAC) and previously served as the Scientific Director of the Institute of Population & Public Health at the Canadian Institutes of Health Research (CIHR). The views expressed in this article are those of the authors and do not necessarily reflect those of PHAC, CIHR or the Government of Canada.*

2. Please provide separate figure files in .tif or .eps format and removed from the manuscript file.

- *We have now uploaded figure files in .tif format*

3. Figure 3: please (a) provide a direct link to the base layer of the map used and ensure this is also included in the figure legend; (b) provide a link to the terms of use / license information for the base layer. We cannot publish proprietary or copyrighted maps (e.g. Google Maps, Mapquest) and the terms of use for your map base layer must be compatible with our CC-BY 4.0 license.

If your map was obtained from a copyrighted source please amend the figure so that the base map used is from an openly available source. Alternatively, please provide explicit written permission from the copyright holder granting you the right to publish the material under our CC-BY 4.0 license.

Please note that the following CC BY licenses are compatible with PLOS license: CC BY 4.0, CC BY 2.0 and CC BY 3.0, meanwhile such licenses as CC BY-ND 3.0 and others are not compatible due to additional restrictions.

If you are unsure whether you can use a map or not, please do reach out and we will be able to help you. The following websites are good examples of where you can source open access or public domain maps:

* U.S. Geological Survey (USGS) - All maps are in the public domain. (http://www.usgs.gov)

* PlaniGlobe - All maps are published under a Creative Commons license so please cite “PlaniGlobe, http://www.planiglobe.com, CC BY 2.0” in the image credit after the caption. (http://www.planiglobe.com/?lang=enl)

* Natural Earth - All maps are public domain. (http://www.naturalearthdata.com/about/terms-of-use/)

- *The base layer of the map used in Figure 3 was obtained from © OpenStreetMap, which is licensed under an Open Database License by the OpenStreetMap Foundation (CC BY-SA 2.0) and can be accessed at this link:*

https://www.openstreetmap.org. More details on terms of use and license information can be found here: https://www.openstreetmap.org/copyright. We have now added this information to the figure legend.

Reviewer #1 Comments

This is an interesting piece using the quasi-experimental analysis to evaluate the impact on transmission of COVID-19 by closure of national borders. While not mentioned, it could also be noted that the piece is timely because with new emerging variants of COVID-19, global cases are still on the rise and proper measures and models for public health interventions are required. The authors have also factored in the biases for covid-19 transmission and border closure.

- *Thank you for these comments. We completely agree that the findings remain timely and could contribute to informing the public health response to emerging variants of COVID-19 as well as future public health emergencies of international concern.*

However, there are a few minor areas in which I feel the paper needs some revision:

1. Line 30, Consider removing 'but no' instead use "Still, no".

- *We have now removed the "but no", which implied that the second half of the sentence was at odds with the first half and replaced it with "and no".*

2. Line 38. Replace 'not sufficient' with insufficient.

- *This use of "not sufficient" is intended to invoke causal language (e.g., sufficient but not necessary), so we have chosen to keep this use as-is.*

3. Line 47. Consider deleting 'decisions on'.

- *We have now reworded to "coordinate national decisions on border closures" to make clear that border closure decision-making is conducted at the country level and that a global coordinating mechanism would not overrule national sovereignty.*

4. The authors have talked about effective reproductive number. As per Cori et al 2013 and Fraser C 2007, the effective reproduction number can be defined in 2 ways: instantaneous reproductive number or as the case reproductive number. Kindly elaborate on which of the two you have considered. I believe that you are considering instantaneous reproductive number but mention this in the text as well.

- *The reviewer is correct in stating that we calculated the instantaneous reproductive number and have now clarified this in the manuscript in the second paragraph of the Methods section.*

5. Line 144: Figure 2 is cited before figure 1 (line 151). Kindly correct this.

- *Thank you for identifying this error, which has now been addressed by exchanging the placement of the two figures.*

6. Can you elaborate on the impact of international border closures for only the top 5 hit nations like USA, India, UK, France etc. in 2021 (the second wave) in a short paragraph in discussion?

- *We have acted on this helpful suggestion in two sections of the discussion. First, we have added detail and four new citations on border closures' impacts on unemployment, higher costs of goods and services, and a historic reduction in growth of economic growth across the world in line 277. By more explicitly detailing these negative consequences, we believe our call to seek less restrictive alternatives whenever possible is made stronger. We have also added a statement to line 320 referencing the failure of targeted border closures enacted in response to the emergence of the Omicron variant of COVID-19 to prevent its rapid global dissemination. Although this second wave is out of scope of this particular study, it does provide valuable context for the interpretation of our findings.*

7. What could be the reason that no major disparities were observed between high-income vs low- and middle-income countries? please shed some light on it.

- *Although this study was not designed to explore the presence or absence of disparities in border closure effectiveness between high-income vs low- and middle-income countries, we have now added brief comment on this observation in the discussion in line 267.*

8. Line 184-190- Out of the countries that had adverse effects of border closures, how frequent the travel is from and to these countries? also, were these countries showed similar outcomes in the following ravel bans in other covid-19 waves as well.

- *We have now added a reference and summary statistics from the International Air Transport Association to the discussion section, which indicates that during our study period a 60-75% reduction in airline travel was observed in every region of the world. For more detailed information, the number of airline passengers for every country analyzed in this study is made available in the supporting open-access dataset.*

9. The biases involved with border closures and Rt has been mentioned properly in the text.

- *We took great care to detail the limitations of our study in the Discussion section and greatly appreciate this acknowledgement.*

10. Lines 303-306. Consider rephrasing to "This study presents the first quasi-experimental analysis of border closures' impacts on viral transmission during a pandemic, taking advantage of an unprecedented natural experiment."

- *Done*

11. Lines 307-311- add 'a' before greater proportion.

- *Done*

12. Lines 311-313- add 'the' before 'threat', 'feasibility' and availability.

- *Done*

13. Line 317. Consider removing 'that were' after government.

- *We have opted to keep this sentence as-is to make clear that these are past government decisions during the 2020 study period.*

Reviewer #2 comments:

The research work described in this paper is commendable considering the time, energy and quantum of work input in this work. The manuscript is technically sound, systematic and meet PGPH publication criteria. The analysis is sound and all needed supporting information have been made available. However, there are a few comments and questions to be attended to by the authors.

- *We thank the reviewer for their helpful suggestions on ways to improve the manuscript, as well as their supportive comments on the quality and merit of our study.*

Introduction: Please kindly provide a global picture of COVID-19 within the first 22 weeks of the pandemic while supporting your literature with figures.

- *Although we currently have a separate manuscript providing a much more detailed investigation of the global picture of COVID-19 during the first 22 weeks of the pandemic under review with another journal, we have provided more context in the introduction as suggested by the reviewer. To do this, we have added a new sentence to the final paragraph of the introduction and moved our figure depicting the evolution of border closures, global daily incidence, R_t, and COVID-19 testing to this section to provide readers with a better understanding of the global situation at this point of the pandemic.*

Methodology: Line 75. Please what criteria was used in the selection of the 166 countries? Please state the inclusion criteria in the main work. What sampling method and technique informed your selection of the 166 countries? Please provide a justification for your choice of the sampling method.

- *We included every country for which COVID-19 incidence data could be obtained from Johns Hopkins University Center for Systems Science and Engineering (CSSE) and for which we could identify covariates relating to country characteristics, economic factors, gender parity, health indicators, and policy control indicators (Table S1; Dataset S1). This information has now been added to the main manuscript in the Methods section rather than only in the Supporting Information file.*

Any justification for limiting your quasi-experimental study to the first 22 weeks of the COVID-19 Pandemic?

- *The decision to limit our study to the first 22 weeks of the COVID-19 pandemic was driven by considerations related to our quasi-experimental methods as well as global border closure trends. Our analysis plan (Dataset S4) was developed once our team determined that new border closures had begun to stall in May 2020, as observed in Figure 2. Because interrupted time series analysis can be performed on a balanced dataset with a minimum of 12 data points (Zhang, 2011), adding our standard lag time of five days to the final country-specific border closure on May 27, 2020 led us to the 22-week mark. Even with these considerations, we conducted robustness checks to ensure that our choice of time period did not impact our results. As observed in Table S8, censoring our interrupted time-series results to 45 days before and after the two intervention points does not meaningfully change the overall results, giving us confidence that our results are robust to this study design choice. These details have been added to the detailed description of our methods in the Supplementary Materials.*

Zhang, F., Wagner, A. K., & Ross-Degnan, D. (2011). Simulation-based power calculation for designing interrupted time series analyses of health policy interventions. Journal of Clinical Epidemiology, 64(11), 1252–1261. https://doi.org/10.1016/j.jclinepi.2011.02.007

Line 243-244: Please operationally define 'total' and 'targeted' at their first appearance in the text.

- *Total and targeted border closures are defined in the first sentence of the manuscript (lines 49-54)*

Line 240-246: Findings well discussed but please kindly relate your findings to the findings of other studies and determine whether they share similarities or dissimilarities.

- *To ensure we have remained up to date, we re-reviewed literature and incorporated relevant new studies that were published after the original submission of our article in the introduction (line 71) and discussion (line 285). Since the existing body of evidence is mixed, our finding aligns with the majority of mathematical models and the largest systematic review, but differ from a recent quasi-experimental study. The reasons for this divergence are explored in more detail in the discussion section.*

Line 263- 264: Please kindly cite an intext reference for that statement since that doesn't seem to reflect a finding from your study.

- *This statement is one of the primary findings of our study and is based on our own analysis. For supporting information, please reference line 161: "Both the level (2.36; CI: 1.18 – 3.53) and slope (0.10; CI: 0.04 – 0.16) of R_t significantly increased as compared to the underlying time trend after the first wave of targeted border closures on February 5, 2020 (Table 1). In contrast, there was a significant drop in the level (-0.62; CI: -1.05 – -0.19) of the global R_t following the wave of total border closures on March 19, 2020, as compared to the first wave of targeted closures.", as well as Table 2 for similar results based on coarsened exact matching.*

Line 288: Please kindly check the font size used

Line 294: Please check and correct the font and size.

- *We have ensured that the font size used in lines 288 and 294 is consistent with the surrounding text.*

Results: Please kindly provide a caption or label for figure 1

- *The caption for Figure 1 can be found on line 95.*
