# Supplementary material for: Quasi-experimental evaluation of national border closures on COVID-19 transmission
Source: PLOS Glob Public Health. 2023 Feb 28;3(2):e0000980. doi: 10.1371/journal.pgph.0000980 (PMC10021705; doi:10.1371/journal.pgph.0000980)
Supplement: Attachment — Submitted filename: Evaluation Manuscript R2 cover letter (2023-01-09).pdf [file pgph.0000980.s007.pdf]

YORK
UNIVERSITÉ
UNIVERSITY

GLOBAL STRATEGY LAB

Dahdaleh Institute for Global Health Research

DAHDALEH BUILDING, 2120
4700 KEELE ST
TORONTO ON
CANADA M3J 1P3
T 416 736 2100

www.globalstrategylab.org

January 9, 2023

Dear colleagues,

Enclosed please find the final edits requested for our provisionally manuscript accepted titled 'Quasi-experimental evaluation of national border closures on COVID-19 transmission'. Each of the following points have now been addressed in order to strengthen the manuscript prior to publication:

1. The role of mathematical models versus observational data.

- The reference to mathematical models has been removed from the abstract.
- A new sentence and reference have been added to the introduction describing the contributions of using real-world data while acknowledging that mathematical models can be valuable during the early stages of a public health event (especially when real-world data are scarce).

2. The dependent variable, R_t.

- A new sentence has been added to the methods section acknowledging potential implications of limited testing supplies early in the pandemic and different countries' approaches to count cases on the calculation of R_t.

3. Total border closures banning vs. targeted border closures.

- The abstract has now been edited to better emphasize differences in the findings between total and targeted border closures.
- Although it is harder to evaluate the impact of targeted border closures due to their uneven implementation, we did analyze the impact of which countries were targeted in the meta-regression and CEM by pulling information on each closure's target countries from the annex of the Oxford data, verifying it, and supplementing it with our own research. This has now been made clearer in the methods section to prevent any future misunderstanding. By evaluating the actual implementation of often mistargeted partial border closures, we are evaluating the real-world effectiveness rather than hypothetical efficacy.

4. Figure 1 y axis.

- The y axis title in Figure 1 has now been edited to this improved wording

5. Linear regression model methods.

- An expanded explanation and new reference have now been added to clarify why linear models provide more conservative and directly comparable outcomes compared to exponential or logarithmic regression models in the methods.

Best,

Mathieu J.P. Poirier, PhD MPH CPH
Steven J. Hoffman, PhD MA JD LLD
on behalf of the study authors
